# Targeting coronaviral inflammation: aptamer-based strategies for emerging threats

**DOI:** 10.1038/s41392-025-02570-8

**Published:** 2026-02-13

**Authors:** Yongyun Zhao, Gang Yang, Zhaoyong Zhang, Mingfeng Xie, Junnan Liu, Yiran Cheng, Yabin Zhang, Xinyu Zhang, Yuchun Wang, Duhan Ma, Longteng Tang, Wei Li, Yanxin Huang, Yongli Bao, Jincun Zhao, Xu Song, Fengming Luo, Huajing Wan

**Affiliations:** 1 Department of Respiratory and Critical Care Medicine, State Key Laboratory of Respiratory Health and Multimorbidity, West China Hospital; Department Center for Functional Genomics and Bioinformatics, College of Life Science, Sichuan University, Chengdu, Sichuan PR China; 2 National Engineering Laboratory for Druggable Gene and Protein Screening, College of Life Science, Northeast Normal University, Changchun, Jilin PR China; 3 Key Laboratory of Respiratory Disease, Guangzhou Institute of Respiratory Disease, The First Affiliated Hospital of Guangzhou Medical University, Guangzhou, Guangdong PR China

**Keywords:** Nucleic-acid therapeutics, Infectious diseases

## Abstract

Coronaviruses have repeatedly emerged in recent years, causing significant and ongoing threats to global public health. The development of therapeutic agents and strategies capable of responding to future outbreaks caused by emerging coronavirus variants remain an ongoing priority. Here, we engineered a single-stranded DNA aptamer (NApt8-3) that selectively binds to the conserved nucleocapsid (N) protein shared among multiple coronaviruses, including SARS-CoV-2 (wild-type, beta, omicron variant), SARS-CoV, MERS-CoV, HCoV-OC43 and HCoV-229E, and strongly inhibits N protein-induced inflammatory cytokine expression. Mechanistically, NApt8-3 effectively binds to the N protein and blocks its interaction with the NLRP3 inflammasome, a key mediator of coronavirus-induced inflammation. To enable intracellular delivery and evaluate its therapeutic potential, we developed a proof-of-concept anti–SARS-CoV-2 agent—circSASON, a circular trivalent aptamer–antisense oligonucleotide (ASO) chimera—combining NApt8-3, an antispike protein aptamer, and an ASO that silences the N gene. In vitro experiments demonstrated that circSASON effectively inhibits SARS-CoV-2 replication and suppresses N protein-induced cytokine expression in host cells. The intranasal administration of circSASON significantly decreased the level of SARS-CoV-2 and alleviated SARS-CoV-2-induced pulmonary inflammation and inflammatory cytokine expression in mice. Therefore, our findings highlight NApt8-3 as a broad-spectrum anti-inflammatory agent that targets the conserved coronavirus N protein. The therapeutic design strategy employed, together with the N aptamer developed in this study, may offer a framework for the rapid development of treatments to combat future pandemics caused by emerging coronavirus variants.

## Introduction

Coronaviruses are a class of single-stranded RNA viruses that have posed significant threats to global health in recent decades, including severe acute respiratory syndrome coronavirus 2 (SARS-CoV-2), severe acute respiratory syndrome coronavirus (SARS-CoV) and Middle East respiratory syndrome coronavirus (MERS-CoV). Most notably, SARS-CoV-2 has caused 776.8 million confirmed infections and over 7 million confirmed deaths worldwide from the start of the pandemic until November 10,2024.^1,2^ These coronaviruses not only cause acute respiratory syndrome but also trigger systemic inflammation and cytokine storms, resulting in high morbidity and mortality.^3–5^ In addition to acute complications, studies have shown that viral proteins and/or RNA may persist in the host for an extended period, a phenomenon particularly evident in SARS-CoV-2 research. The persistent presence of viral components may contribute to prolonged immune activation and chronic inflammation, potentially contributing to the post-acute sequelae of coronavirus disease 2019 (COVID-19).^6–9^ Although these coronavirus epidemics have been brought under control, the high mutation rate of coronaviruses presents high potential for future recurrent outbreaks. Therefore, the development of therapeutics that have broad-spectrum anti-inflammatory activity to alleviate diseases caused by coronavirus infections remain a critical research priority in drug development.

Currently, several categories of direct-acting anti-coronavirus drugs have been developed: one category includes spike (S) protein-neutralizing antibodies, such as sotrovimab and Bertilimumab, which prevent SARS-CoV-2 entry into host cells. However, owing to the high variability of the S protein among coronaviruses,^10^ these drugs cannot be used as broad-spectrum anti-coronavirus drugs to control emerging outbreaks. Another category consists of small-molecule inhibitors that target coronavirus protease (Nirmatrelvir), RNA-dependent RNA polymerase (Remdesivir)^11^ or both (Paxlovid),^12^ thereby disrupt viral replication. However, these drugs have a narrow treatment window and show limited efficacy in severe cases, and obvious toxic side effects, including liver and kidney injury, hypertension and dizziness. Their antiviral activity also dependents on timely administration during early viral replication, which restricts their applicability in patients who present late or already exhibit hyperinflammatory responses. In addition, two main classes of immunomodulators are commonly used to mitigate excessive inflammation and prevent cytokine storms. The first class includes nonspecific immune modulators, such as cyclosporine and glucocorticoids, which broadly suppress immune responses. The second class comprises targeted immunomodulators, which specifically inhibit proinflammatory cytokines or signaling pathways. Examples include IL-6 receptor antagonists (e.g., tocilizumab and sarilumab), IL-1 receptor antagonists (e.g., anakinra), TNF-α inhibitors (e.g., adalimumab and infliximab), and JAK/STAT pathway inhibitors (e.g., ruxolitinib and baricitinib). Nonetheless, these methods are limited by a lack of coronavirus-specific activity, a risk of secondary infection, off-target effects, etc. Recent studies have demonstrated that the N protein is one of the most abundant viral proteins identified in virus-infected cells. It is highly conserved across coronaviruses and plays an essential role in viral replication, assembly, and inflammation, making it a promising target for broad-spectrum antiviral drug development.^13–19^ Thus, therapeutics targeting the N protein could provide both antiviral and anti-inflammatory benefits, offering a broad-spectrum strategy for future coronavirus outbreak control.

Antibodies and small-molecule inhibitors have been widely utilized in the development of traditional therapeutics for coronavirus-associated diseases. However, the time-consuming screening process limits their ability to meet the urgent need for drug development in response to newly emerged viral mutants. In contrast, aptamers offer a more rapid and flexible alternative. Aptamers are structured, short single-stranded DNA/RNA sequences that can bind to their targets with high affinity and specificity. Aptamers can be rapidly screened via the systematic evolution of ligands via exponential enrichment (SELEX) technology. With advantages such as a short development cycle, low immunogenicity, easy reconfigurability and fast adaptability to emerging viral mutants, aptamers represent promising tools for virus detection, trapping or neutralization. Since the outbreak of SARS-CoV-2, a wide range of aptamers that target coronavirus S proteins, glycan shields, virus-associated enzymes, and N proteins have been identified.^20–24^ Among these, several high-affinity aptamers targeting the N protein have been developed, recently.^25^ However, because efficient intracellular delivery—particular to specific tissues or infected cells-remains a major obstacle, their anti-inflammatory effects against coronaviruses in vivo and their potential as reliable therapeutic agents for controlling coronavirus-induced pulmonary diseases are still unknown.^25^ This limitation underscores the need for delivery strategies capable of ensuring adequate intracellular uptake in relevant target cells.

Here, we developed an N protein aptamer (NApt8-3) via our previously reported technique,^26^ which blocks the interaction between N protein and the NLRP3 inflammasome and exhibits broad-spectrum anti-inflammatory effects to combat various pathogenic coronaviruses, including SARS-CoV-2 (WT), SARS-CoV-2 (beta, B.1.351), SARS-CoV-2 (Omicron, BA.2.3), SARS-CoV, MERS-CoV, HCoV-OC43, and HCoV-229E. Furthermore, to assess the anti-inflammatory and therapeutic potential of the aptamer NApt8-3, we designed a circular trivalent aptamer-ASO chimera (circSASON) by combining S- and N-binding aptamers with an N antisense oligonucleotide, enabling specific delivery into virus-infected cells and exhibit synergistic inhibitory effects on N protein. The intranasal administration of circSASON significantly decreases the level of SARS-CoV-2 and alleviates SARS-CoV-2-induced pulmonary inflammation in mice.

## Results

### Identifying a high-affinity DNA aptamer (NApt8-3) that targets the N protein of coronavirus

**Fig. 1:**
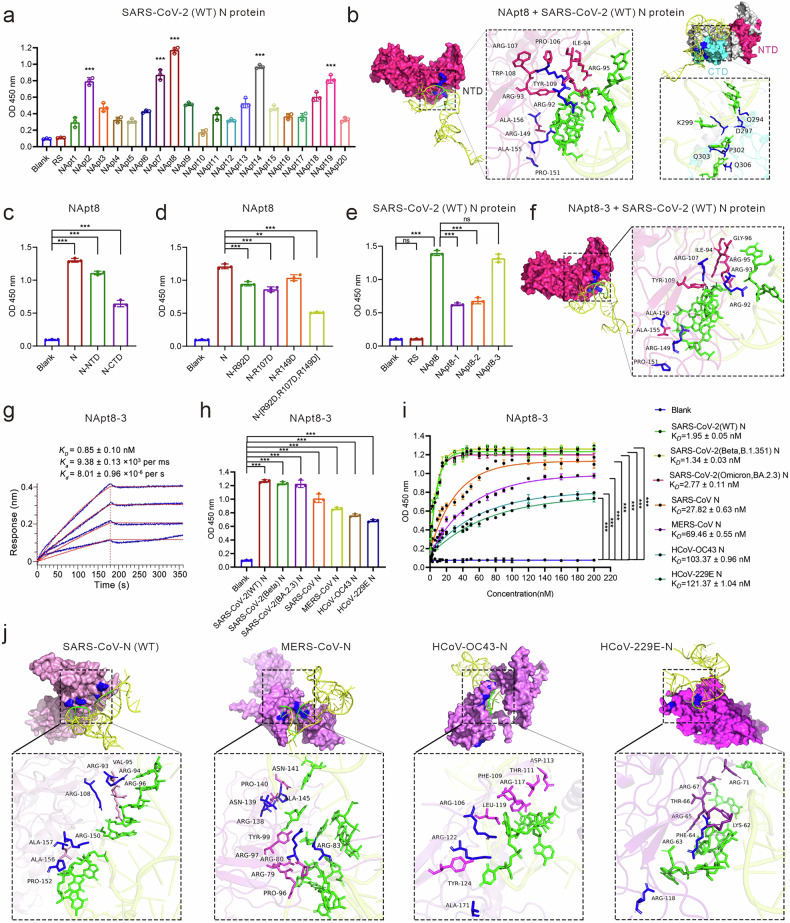
Identification of a high-affinity DNA aptamer targeting the N protein of coronavirus (NApt8-3). **a** ELISA analysis used to assess the binding of biotin-labeled candidate DNA aptamers (200 nM) to precoated SARS-CoV-2 N protein (100 ng/well). The optical density (OD) at 450 nm represents the binding signal. RS (random sequence) and the blank (without any treatment) were used as negative controls. **b** Molecular docking simulation of NApt8 and SARS-CoV-2 N protein binding via Discovery Studio software. The nucleobases of NApt8 (sequence provided in Table S2) are labeled in green. Left: docking with the NTD; Right: docking with the CTD; magenta and cyan represent the NTD and CTD, respectively. **c** ELISA analysis was used to assess the binding of biotin-labeled NApt8 (200 nM) to precoated (100 ng/well) N protein (N), the NTD domain of the N protein (N-NTD) or the CTD domain of the N protein (N-CTD) on microplates. Blank: noncoated microplates. **d** ELISA analysis of the ability of a biotin-labeled aptamer (200 nM) to bind the mutated N protein (100 ng/well) precoated on microplates. The mutated N protein: arginine (R) was mutated to aspartic acid (D). **e** ELISAs to assess the binding performance of the biotin-labeled truncated aptamer (200 nM) to N protein (100 ng/well) precoated on microplates. NApt8-3, NApt8-2 and NApt8-1 (sequences provided in Table S2) represent truncated variants generated by deleting nonessential sequences at the 5’ end, 3’ end and both ends, respectively. **f** Molecular docking simulation of NApt8-3 and SARS-CoV-2 (WT) N protein binding via Discovery Studio software. **g** Biolayer interferometry (BLI) assay to determine the kinetic binding parameters of the aptamer NApt8-3 to immobilized N proteins. **h** ELISAs were used to assess the binding performance of biotin-labeled NApt8-3 (200 nM) to the N protein of various coronaviruses (SARS-CoV-2 and its variants, SARS, MERS, HCoV-OC43, and HCoV-229E). **i** ELISA analysis of the kinetics of the binding of the aptamer NApt8-3 to the N protein of various coronaviruses, including SARS-CoV-2 variants (WT, Beta B.1.351, and Omicron BA.2.3), SARS-CoV, MERS-CoV, HCoV-OC43, and HCoV-229E. **j** Molecular docking simulation of NApt8-3 to the N proteins of SARS-CoV, MERS-CoV, HCoV-OC43, and HCoV-229E via Discovery Studio software. The N protein is shown with residue names. Amino acids shown in magenta represent conserved residues across coronaviruses that interact with NApt8-3, while blue denotes residues uniquely involved in binding in specific viral strains. Green represents the nucleobases of NApt8-3. All the error bars indicate standard deviations (*n* = 3). All the *P* values were calculated via one-way ANOVA. ***P < 0.001

To identify N protein-specific aptamers, we used the full-length SARS-CoV-2 N protein as bait to perform aptamer screening via the microwell SELEX system illustrated in Supplementary Fig. 1a. Briefly, a microwell plate was coated with the SARS-CoV-2 N protein (full length) and incubated with a synthetic ssDNA library for SELEX. To reduce nonspecific binding, after 6 rounds of selection, the total protein in human lung epithelial cells (A549) was used as the negative target to conduct counterselection. After an additional 12 rounds of selection, the final pool of aptamer products was assessed via next-generation sequencing, and NApt8, the most abundant ssDNA, was selected as the candidate N protein aptamer (Fig. 1a). We analyzed the 3D structure of NApt8 via an RNA composer and performed molecular docking simulations, which suggested that the green-labeled nucleobases of NApt8 bind to both the N-terminal domain (NTD, aa: 44–174) and the C-terminal domain (CTD, aa: 260–340) of the N protein (Fig. 1b). In the NTD, NApt8 was positioned in a positively charged pocket with close contacts with R92, R107, and R149 (Fig. 1b). ELISAs confirmed these interactions and revealed a greater affinity for the NTD than for the CTD (Fig. 1c). To validate the NTD interface, site-directed mutagenesis of positively charged R92, R107 and R149 to negatively change aspartic acid markedly reduced the Napt8-N protein interaction, demonstrating that these residues are critical for binding (Fig. 1d). To facilitate the future development of anticoronaviral therapeutics based on NApt8, we generated its truncated variants, designated NApt8-3, NApt8-2 and NApt8-1, by removing nonessential sequences at the 5′, 3′ or both terminal sequences, respectively. ELISA, flow cytometry, and fluorescence imaging experiments consistently demonstrated that NApt8-3 exhibited the highest affinity for the N protein, comparable to that of NApt8. However, significantly decreased N protein binding affinity was observed for NApt8-1 and NApt8-2. These data indicate that the 3’ terminal sequence of NApt8 is essential for N protein binding (Fig. 1e, Supplementary Fig. 1b). The results of the molecular docking simulations of the aptamer NApt8-3 were highly similar to those of NApt8 (Fig. 1f). The binding kinetics were measured via a biolayer interferometry (BLI) assay with purified and immobilized N proteins. The dissociation constant (Kd) for the binding of NApt8-3 to the N protein was 0.85 nM (Fig. 1g), demonstrating an order of magnitude greater affinity than typical aptamers.^27,28^ Furthermore, NApt8-3 exclusively binds to the SARS-CoV-2 N protein, with no detectable interaction observed with other viral structural proteins (spike, envelope or membrane proteins) or with host cellular proteins in the lysates derived from Calu-3, A549, or HEK293T cells (Supplementary Fig. 1d). This binding specificity suggests a reduced likelihood of off-target effects of Napt8-3.

To validate whether NApt8-3 could be used as a broad-spectrum N protein binding aptamer, the binding affinity of NApt8-3 for the full-length N proteins of other coronaviruses was evaluated. The results demonstrated that the aptamer NApt8-3 could bind to N proteins of other coronaviruses, including SARS-CoV-2 (WT, Beta B.1.351, Omicron BA.2.3), SARS-CoV, MERS-CoV, HCoV-OC43 and HCoV-229E (Fig. 1h). NApt8-3 has low nanomolar Kd values against the coronavirus N protein, with Kd values ranging from 1.95 to 121.37 nM (Fig. 1i). The NTD domain between SARS-CoV-2 variants is highly conserved, with a similarity of up to 95.72%. Consistent with this, no difference in their affinities for NApt8-3 was detected. The results from the molecular docking simulations also confirmed that the binding of NApt8-3 to the NTD domain is highly conserved among these coronaviruses (Fig. 1j, Supplementary Fig. 1e). Taken together, these results demonstrate that NApt8-3 has binding affinity for the N protein from different coronaviruses and could be used as a broad-spectrum N protein-binding aptamer.

### Broad-spectrum inhibitory effects of NApt8-3 on the inflammatory response induced by the N protein derived from various coronaviruses

**Fig. 2:**
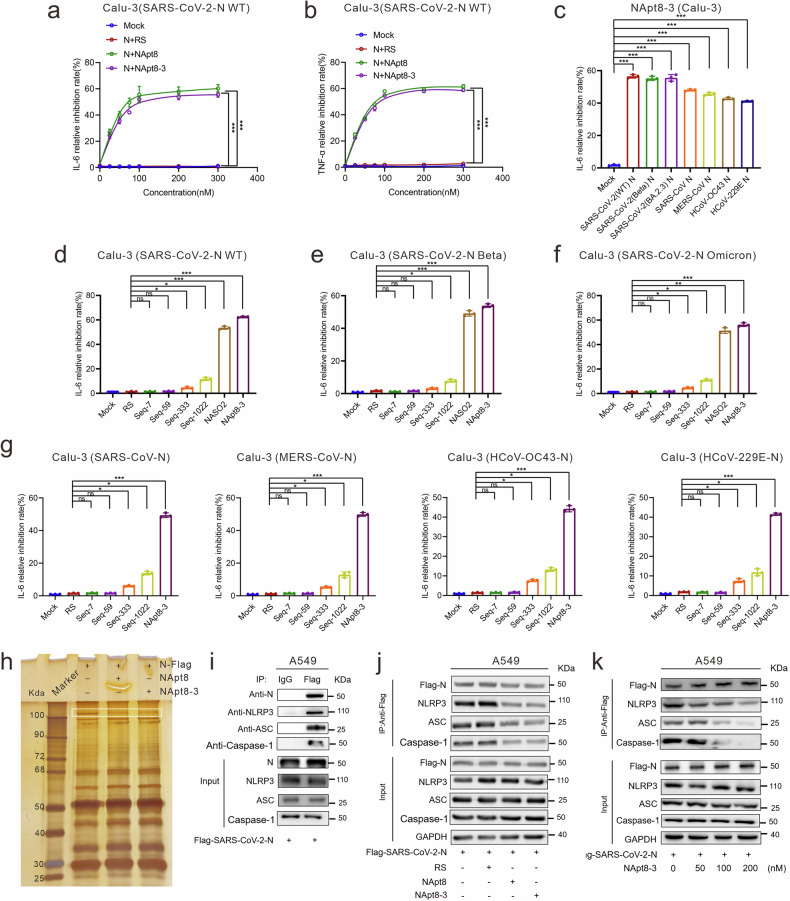
Broad-spectrum inhibitory effects of NApt8-3 on the inflammatory response induced by the N protein derived from various coronaviruses. **a**, **b** qRT‒PCR analysis of the dose-dependent inhibitory effects of NApt8 and NApt8-3 on the expression of cytokines (IL-6 and TNF-α) in Calu-3 cells transfected with a plasmid expressing the N protein. Mock: cells transfected with the N-encoding plasmid but without a random DNA sequence (RS) and aptmer treatment. RS: random sequence. The mRNA level of actin was used for normalization. The methods used to calculate the relative inhibition are described in the Materials and Methods. **c** qRT‒PCR analysis of the expression of cytokines (IL-6) in Calu-3 cells cotransfected with NApt8-3 together with plasmids expressing the N protein of various coronaviruses and SARS-CoV-2 variants (WT, Beta B.1.351, Omicron BA.2.3), SARS, MERS, HCoV-OC43, and HCoV-229E). **d**‒**g** qRT‒PCR analysis of the expression of cytokines (IL-6) in Calu-3 cells cotransfected with aptamers (NApt8-3, seq-7, seq-59, seq-333, seq1022, NASO2) and plasmids expressing the N protein of different coronaviruses. **h** Silver-stained SDS‒PAGE was used for coimmunoprecipitation with anti-Flag antibodies to identify proteins that interact with N-Flag in the lysates of A549 cells transfected with N-Flag-expressing plasmids subjected to the indicated treatments. The white box denotes the differentially expressed protein band (100 kDa). **i**. Coimmunoprecipitation was performed via the use of anti-FLAG antibodies to confirm the interaction of the N protein and NLRP-3 in the lysates of A549 cells. Cell lysates (40 μg) were used as inputs. **j** Coimmunoprecipitation using anti-NLRP3 antibodies was used to confirm the interaction between the N protein and NLRP-3 in the lysates of A549 cells subjected to the indicated treatments. Cell lysates (40 μg) were used as inputs. **k** NApt8-3 inhibits the NLRP3-N protein interaction in a dose-dependent manner. All the error bars indicate standard deviations (*n* = 3). *P* values were calculated via one-way ANOVA. *P < 0.05; **P < 0.01; ***P < 0.001

To validate the effects of NApt8-3 on the SARS-CoV-2 N protein-induced inflammatory response, we cotransfected human lung epithelial cells (Calu-3) with a plasmid encoding the SARS-CoV-2 N protein and NApt8-3. The inflammatory response was assessed by measuring the relative mRNA levels of IL-6 and TNF-*α*, which were normalized to the level of actin mRNA. We found that NApt8-3 remarkably inhibited N protein-induced IL-6 and TNF-α expression in Calu-3 cells (Fig. 2a and b, Supplementary Fig. 2a), and the inhibition efficiency was highly consistent with that of NApt8. Similarly, the anti-inflammatory activity of NApt8-3 was also observed in A549 cells (Supplementary Fig. 2b). To assess whether NApt8-3 can inhibit the inflammatory response induced by the N protein from other coronaviruses, Calu-3 cells were cotransfected with plasmids encoding the N protein of different coronaviruses and NApt8-3, and NApt8-3 markedly inhibited N protein-induced IL-6 production in Calu-3 cells (Fig. 2c). This result was highly consistent in A549 cells, and the expression levels of the different N proteins were tightly controlled and comparable (Supplementary Fig. 2c–e). We chose the optimum ASO, which targeted the N gene (NASO2), as the positive control (Supplementary Fig. 2f and g). Seq-7, Seq-59, Seq-333, Seq-1022 and NASO2 can specifically target the SARS-CoV-2 N protein.^25,28^ We compared the anti-inflammatory effects of NApt8-3 and found that NApt8-3 had the greatest anti-inflammatory effect on SARS-CoV-2 N protein-induced IL-6 expression. The inhibition rate of NApt8-3 is approximately 41.7–60%, which is comparable to that of NASO2 for N gene silencing (Fig. 2d–f). However, the anti-inflammatory effects of Seq7/59 have not been detected, and those of Seq1022 and Seq333 were significantly lower than those of NApt8-3 (Fig. 2g).

To investigate the mechanism underlying the inhibitory effects of NApt8-3 on N protein-induced inflammation, we performed immunoprecipitation (IP) assays to identify N protein binding partners whose interactions may be disrupted or modulated by NApt8-3. Compared with that of the controls, the intensity of the protein band at approximately 100 kDa was significantly lower in the Napt8-3-treated group and was subsequently subjected to LC‒MS/MS analysis. Among the detected protein peptides, NOD-like receptor thermal protein domain-associated protein 3 (NLRP3) exhibited a high reliability score (Fig. 2h, Supplementary Table 1). Consistent with previous studies showing that the interaction of the N protein with NLRP3 can recruit ASC and Caspase-1 and activate cytokine (IL-1β and IL-18) production,^16,29,30^ our co-IP experiments demonstrated that the N protein binds to NLRP3, ASC and caspase-1 (Fig. 2i). NApt8-3 significantly inhibited the binding of the N protein to NLRP3, ASC and caspase-1 in a dose-dependent manner (Fig. 2j and k, Supplementary Fig. 2h) and inhibited N protein-induced IL-1β and IL-18 expression in Calu-3 cells (Supplementary Fig. 2i). Molecular docking simulations revealed that NApt8-3 and NLRP3 bind to both the CTD and NTD of the SARS-CoV-2 N protein (Fig. 1b, Supplementary Fig. 2j). ELISAs confirmed the interactions of NLRP3 with both the CTD and NTD of the SARS-CoV-2 N protein, which displayed a greater affinity for the CTD than for the NTD. Furthermore, competition ELISA revealed that NApt8-3 significantly blocked the interactions among NLRP3-N, NLRP3–N-CTD and NLRP3–N-NTD (Supplementary Fig. 2k). Taken together, these results demonstrate that NApt8-3 has broad-spectrum anti-inflammatory effects on *coronaviruses*, including SARS-CoV-2 (WT, Beta B.1.351, and Omicron BA.2.3), SARS-CoV, MERS-CoV, HCoV-OC43 and HCoV-229E, in vitro. NApt8-3 has an anti-inflammatory function through the NLRP3 signaling pathway.

### Engineering a trivalent-aptamer therapeutic for SARS-CoV-2 via NApt8-3

**Fig. 3:**
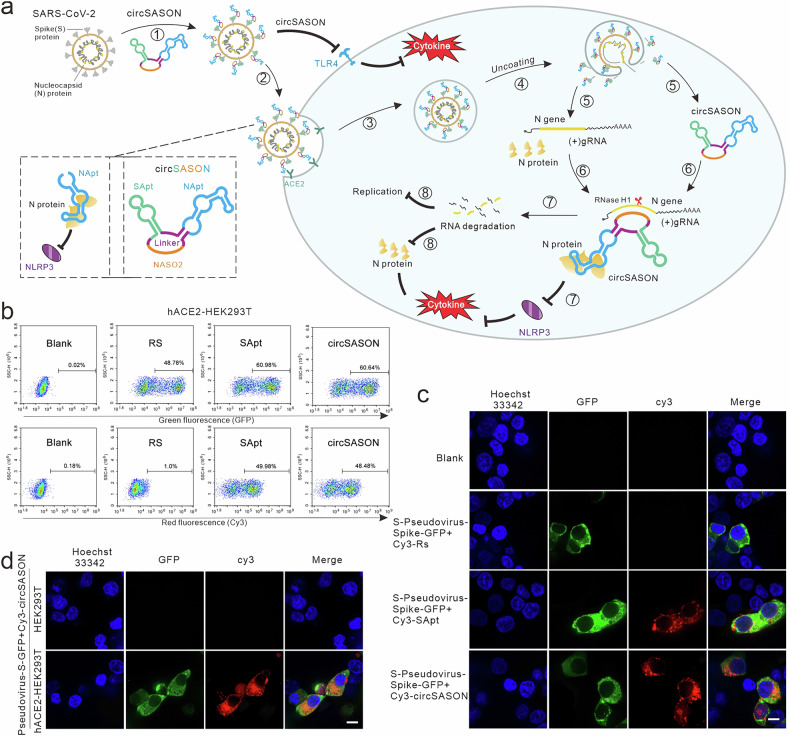
Design and performance of a circular trivalent chimera for SARS-CoV-2 via NApt8-3. **a** Schematic illustration of the principle underlying efficient targeted delivery of circSASON to suppress SARS-CoV-2 (WT) replication and inflammation. CorelDraw was used to generate these images. **b** Flow cytometry analysis of SApt-dependent and SARS-CoV-2 infection-mediated delivery of circSASON into hACE2-HEK293T cells. Blank (without any treatment) and RS were used as negative controls. SApt as a positive control. The upper panel shows the GFP signal from the SARS-CoV-2 Spike (WT) Fluc-GFP pseudovirus. The lower panel displays the Cy3 signal from the Cy3-labeled oligonucleotides (RS, SApt or cirSASON). **c** Representative fluorescence confocal images of hACE2-HEK293T cells infected with SARS-CoV-2 pseudovirus (5 × 10^4^ TCID50 mL^−1^) preincubated with the indicated 200 nM Cy3-labeled oligonucleotides (*n* = 3). S-pseudovirus spike-GFP: SARS-CoV-2 spike (WT) Fluc-GFP pseudovirus. Scale bars represent 10 µm. **d** Representative fluorescence confocal images of HEK293T and hACE2-HEK293T cells incubated with SARS-CoV-2 pseudovirus (5 × 10^4^ TCID50 mL^−1^) and Cy3-labeled circSASON (200 nM). Scale bars represent 10 µm

To deliver NApt8-3 into virus-infected host cells and investigate the value of NApt8-3 in controlling coronavirus-triggered inflammation, we designed an anti-SARS-CoV-2 drug via an aptamer-dependent and viral infection-mediated delivery strategy, as described in our previous paper.^31^ To increase the stability of the drug, T4 ligase was used to construct a circular trivalent aptamer-ASO chimera (circSASON), connecting Napt8-3 (NApt, blue) with a SARS-CoV-2 spike protein aptamer (SApt, green) and an antisense oligonucleotide for N gene silencing (NASO2, orange) (Fig. 3a). The functions of SApt and NASO2 were validated, and the methods were described in our previous paper.^32,33^ As shown in Fig. 3a, when SARS-CoV-2 infects ACE2-positive host cells (step 1), circSASON binds to the S protein of SARS-CoV-2 and suppresses S-induced cytokine production by blocking its interaction with Toll-like receptor 4 (TLR4). Since SApt does not block the S/ACE2 interaction, circSASON can be delivered into susceptible host cells along with SARS-CoV-2 (step 2). After entering the cell, the viral proteins, RNAs and circSASON are released into the cytoplasm of the host cells (steps 3, 4, 5). The NApt module of circSASON binds to the N protein and blocks N/NLRP3 to inhibit N-induced cytokine production (step 7). Moreover, the NASO module specifically binds to N mRNA (step 6), which silences mRNA expression, thus effectively inhibiting SARS-CoV-2 replication (steps 7 and 8). According to the above design principles, the circSASON we developed can be delivered into ACE2-positive host cells with SARS-CoV-2 and subsequently inhibits both viral replication and the activation of the virus-induced host immune response.

To validate whether circSASON could be specifically delivered into SARS-CoV-2-infected hACE2-positive cells, Cy3-labeled circSASON and SARS-CoV-2 spike (WT) Fluc-GFP pseudovirus were incubated together with hACE2-HEK293T cells. Cy3-labeled RS (random sequence) and Cy3-labeled SApt were used as negative and positive controls, respectively. After 48 h of incubation, quantitative flow cytometry analysis (Fig. 3b, Supplementary Fig. 3a) and confocal fluorescence imaging analysis were carried out (Fig. 3c, Supplementary Fig. 3b). The results revealed that both SApt and circSASON were delivered into SARS-CoV-2 pseudovirus-positive hACE2-HEK293T cells. The efficiency of circSASON delivery increased in a viral dose-dependent manner (Supplementary Fig. 3c). In addition, circSASON cannot be delivered into hACE2-negative HEK293 cells under the same experimental conditions (Fig. 3d, Supplementary Fig. 3d). Collectively, these results demonstrate that circSASON can be specifically and efficiently delivered together with SARS-CoV-2 into ACE2-expressing cells.

### Validating the stability and trivalent function of circSASON

**Fig. 4:**
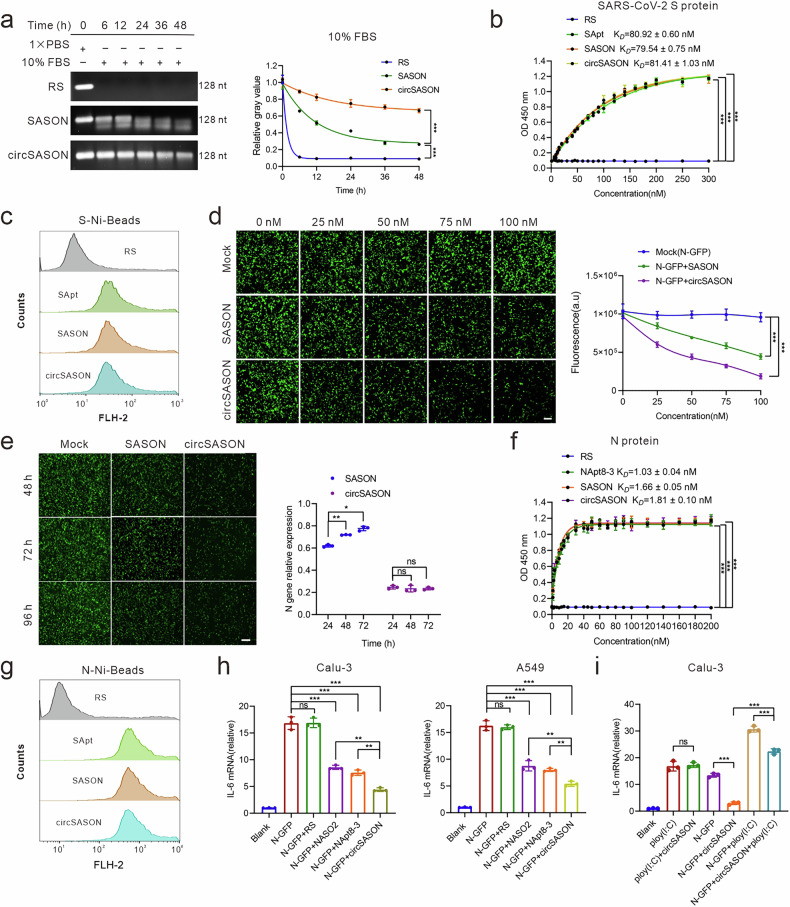
Validation of the stability and trivalent function of circSASON. **a** Stability and quantitative analysis of cirSASON incubated in 10% FBS at 37 °C at different time points. RS and linear SASON were used as controls. The right panel presents the quantitative data for the results shown in the left panel. Nt (nucleotide) denotes the length of single-stranded nucleic acids. **b** ELISA was used to assess the binding performance of biotin-labeled controls and cirSASON to the spike protein. **c** Flow cytometry was used to investigate the binding performance of Cy3-labeled circSASON to the spike protein prebound to Ni beads. **d** Representative fluorescence imaging analysis of the dose-dependent inhibitory effects of circSASON and linear SASON on GFP-N protein expression (*n* = 3). Scale bars represent 50 µm. The right panel shows the quantification of the fluorescence images in the left panel. **e** Representative fluorescence imaging analysis of the time-dependent inhibitory effects of circSASON and linear SASON on N protein expression (*n* = 3). Scale bars represent 50 µm. The right panel shows the quantification of the fluorescence images in the left panel. The *P* values were calculated via two-way ANOVA. **f** ELISA was used to assess the binding performance of biotin-labeled aptamers to the N protein. **g** Flow cytometry was used to evaluate the binding performance of Cy3-labeled circSASON to the spike protein prebound to Ni beads. SApt and SASON were used as positive controls. **h** qRT‒PCR analysis of the relative expression of *IL-6* (to actin) in Calu-3 cells and A549 cells treated as indicated. The *P* values were calculated via one-way ANOVA. **i** qRT‒PCR analysis of the immune response to Poly (I:C) in Calu-3 cells subjected to the indicated treatments. The last sample consisted of Calu-3 cells pretransfected with a plasmid encoding the N-GFP and circSASON (100 nM). The cells were then transfected with poly(I:C) (5 µg mL^−1^) to induce cytokine production again. The *P* values were calculated via one-way ANOVA. All the error bars indicate standard deviations (*n* = 3). **P < 0.01; ***P < 0.001

To validate the stability of circSASON, enzymatic digestion experiments were carried out. Compared with RS and SNASO (linearized circSASON), circSASON presented the strongest enzymatic resistance and maintained its integrity until 24 h (Fig. 4a). To validate whether circSASON preserved the functions of SApt, NASO and NApt8-3, the following experiments were performed. First, the ability of circSASON to bind the S protein was assessed via ELISA and flow cytometry. Both results revealed that the ability of circSASON to bind S proteins was similar to that of SApt and SASON (Fig. 4b and c). Second, to validate N gene silencing activity, circSASON and SASON were cotransfected with a SARS-CoV-2 N-GTP-expressing plasmid into HEK293T cells. The cells were harvested after 48 h for immunofluorescence imaging. The results demonstrated that circSASON exhibited stronger and more sustained inhibitory effects on N gene silencing than did SASON. Moreover, the silencing efficiency of circSASON was dose dependent (Fig. 4d, Supplementary Fig. 4a and b). Furthermore, owing to its high stability, circSASON also effectively silences N gene expression for a long time (72 h) (Fig. 4e). Third, we assessed whether circSASON preserved the function of NApt8-3. ELISAs and flow cytometry revealed that the binding ability of circSASON to N proteins was similar to that of NApt8-3 (Fig. 4f and g). As previously reported, SApt does not have anti-inflammatory activity against N protein-induced inflammation.^32^ Compared with NApt8-3 and NASO, circSASON more strongly suppressed N protein-induced IL-6 expression in Calu-3 and A549 cells (Fig. 4h, Supplementary Fig. 4c). Furthermore, Bliss independence analysis demonstrated synergistic anti-inflammatory activity arising from the combined function of NApt8-3 and NASO (Supplementary Fig. 4d).

Poly (I:C) is a synthetic analog of double-stranded RNA (dsRNA) that is known to activate the NLRP3 inflammasome to induce the production of proinflammatory cytokines. To test the specificity of circSASON for SARS-CoV-2 N-induced NLRP3 signaling, the inhibitory effects of circSASON on IL-6 production induced by Poly(I:C) were also assessed in Calu-3 and A549 cells. The results revealed that circSASON cannot inhibit poly (I:C)-induced IL-6 production. Moreover, when poly(I:C) was transfected into cells in which N-induced inflammation was suppressed by circSASON, the cells reproduced cytokines again (Fig. 4i, Supplementary Fig. 4e). These results suggest that circSASON is a specific inhibitor of SARS-CoV-2-induced inflammation.

### circSASON exhibits strong anti-SARS-CoV-2 efficacy in host cells

**Fig. 5:**
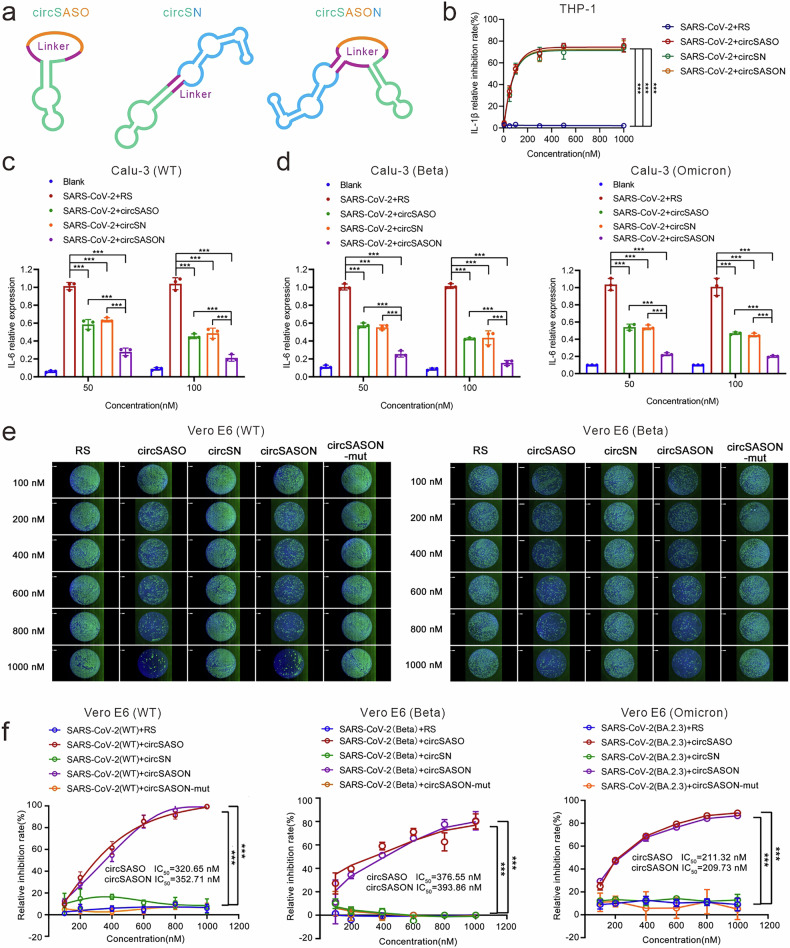
circSASON inhibits SARS-CoV-2 replication and inflammatory cytokine expression. **a** The secondary structure of the three circular chimeras (circSASO, circSN and circSASON) predicted via RNAfold. **b** qRT‒PCR analysis of the expression of IL-6 in Calu-3 cells and A549 cells treated as indicated. mRNA (relative) represents the mRNA expression level normalized to that of actin mRNA. **c**, **d** qRT‒PCR analysis of the inhibitory effects of the three circular chimeras on IL-6 expression in cells 48 h after infection with SARS-CoV-2 variants (WT, Beta B.1.351, or Omicron BA.2.3) (2 × 10^6^ PFU/mL, MOI = 0.01). **e** Immunofluorescence staining assay of SARS-CoV-2 replication in Vero E6 cells incubated with SARS-CoV-2 WT or Beta B.1.351 (2 × 10^6^ PFU/mL, MOI = 0.01) and the indicated circular chimeras for 48 h. Scale bars represent 1 mm. **f** Immunofluorescence (IFA) was used to quantify the intracellular nucleocapsids of SARS-CoV-2 WT, Beta B1.351 or Omicron BA.2.3 (2 × 10^6^ PFU/mL, MOI = 0.01) in Vero E6 cells after 48 h of the indicated treatment. The IC50 values of circSASO and circSASON are indicated in the curves. All the error bars indicate standard deviations (*n* = 3). **b**, **d**, **f** *P* values were calculated via one-way ANOVA. **c**, **d**: *P* values were calculated via two-way ANOVA. ***P < 0.001

To evaluate the therapeutic effects of circSASON against SARS-CoV-2, we compared its anti-inflammatory and antiviral efficacy with two anti-SARS-CoV-2 aptamer chimeras we previously developed: circSASO (a composite agent consisting of SApt and NASO) and circSN (a composite agent consisting of SApt and the aptamer NApt8-3) (Fig. 5a). We transfected circSASON, circSASO or circSN with a SARS-CoV-2 N-GFP-expressing plasmid into cells (A549 and Calu-3), and N protein-induced cytokine expression was measured via qRT‒PCR. Compared with circSASO or circSN, circSASON had significantly stronger inhibitory effects on the expression of SARS-CoV-2 N protein-induced cytokines, including IL-6 and TNF-α (Fig. 5b, Supplementary Fig. 5a). Furthermore, the inhibitory effects of these aptamer chimeras on cytokine expression induced by SARS-CoV-2 variants, including WT, Beta B.1.351 and Omicron BA.2.3, were tested and compared. Compared with circSASO or circSN, circSASON demonstrated much stronger inhibitory effects on cytokine expression induced by SARS-CoV-2 variants (Fig. 5c and d, Supplementary Fig. 5b–d). A time-course experiment with Calu-3 cells was performed to monitor anti-N-induced inflammation in response to treatment with circSASO, circSN, and circSASON over time (Supplementary Fig. 5e). Moreover, Bliss independence analysis demonstrated that circSASON not only silences N gene expression but also antagonizes N-induced inflammation, which synergistically mitigates N-induced inflammatory efficacy (Supplementary Fig. 5f and g).

To compare the antiviral efficacy of circSASON with that of circSASO and circSN, these aptamer chimeras and SARS-CoV-2 (WT) were added to the culture media of Vero E6 cells. RS and the circSASON mutant (a scrambled version of the circSASON sequence, Table S2) were used as negative controls. After 48 h of incubation, immunofluorescence assays of the N protein and IC50 of SARS-CoV-2 variants (WT, Beta B.1.351, Omicron BA.2.3) were performed. As shown, both circSASO and circSASON significantly inhibited active viral replication with similar IC50 values (Fig. 5e and f), which is comparable to that of remdesivir.^34^ Likewise, circSASON and circSASO, but not circSN, remarkably inhibited the replication of SARS-CoV-2 variants (WT, Beta B.1.351, and Omicron BA.2.3) (Fig. 5e and f, Supplementary Fig. 5h). These findings further suggest that circSASON disturbs the replication of SARS-CoV-2 via the silencing of the N gene. Together, these results demonstrate that circSASON exhibits robust anti-inflammatory activity and anti-replication activity against SARS-CoV-2 and its variants.

### circSASON significantly alleviates SARS-CoV-2-induced pulmonary inflammation

To evaluate SARS-CoV-2-mediated circSASON delivery in vivo, BALB/c mice were intranasally administered a mixture of Cy5-labeled cirSASON and SARS-CoV-2 spike (WT) pseudovirus. Two hours, 24 h and 48 h later, the biodistribution of circSASON was analyzed in samples collected from the heart, liver, lung, spleen and kidney. The results (Supplementary Fig. 6a) revealed that circSASON was detected in the liver, lung, kidney and spleen at 2 h, with the highest levels in the lung and liver. By 48 h, circSASON levels markedly decreased in the lung and kidney but increased in the liver. No circSASON signal was detected in the heart at any time point. To evaluate the immunogenicity of circSASON, we measured the mRNA levels of inflammatory cytokines in mice at the 48-h time point after intranasal administration of circSASON. Poly(I:C), known to have strong immunogenicity, was used as a positive control. No significant changes were observed in the expression of IL-6, TNF-α or IFN-γ in either the lung or plasma following circSASON treatment (Supplementary Fig. 6b and c), indicating that circSASON has low immunogenicity.

**Fig. 6:**
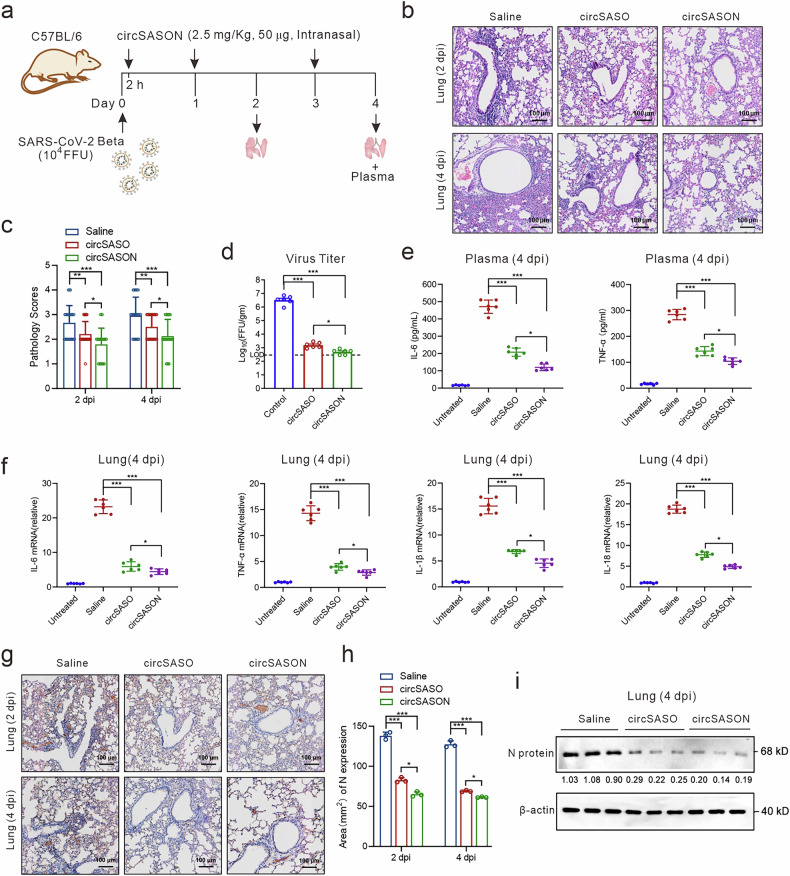
circSASON protects mice against Beta VOC infection. **a** Schematic illustration of the mouse infection experiment. The wild-type mice were intranasally inoculated with 10^4^ FFU of the SARS-CoV-2 beta variant (B.1.351) and treated intranasally with 50 µg (2.5 mg/kg, body weight = 20 g) circSASON, circSASO or vehicle control (0.9% saline) at 2, 24 and 72 h post-infection. **b** Representative H&E-stained lung sections (*n* = 3 mice per group) collected at 2 days post-infection (dpi) and 4 dpi. Scale bars, 100 μm. **c** Comparison of the pathology scores of lungs collected from different treatment groups. **d** Focus formation assay analysis of the viral burden in the lungs of mice (*n* = 6 mice per group) using lung homogenates at 2 dpi. LOD: limit of detection. **e**, **f** qRT‒PCR and ELISA analysis of cytokines in the lung tissue and plasma of mice at 4 dpi (*n* = 6 mice per group), respectively. **g** Representative images of N protein and NLRP3 immunohistochemical staining at 2 dpi and 4 dpi, respectively. Scale bars, 100 μm. **h** Quantitative evaluation of N protein and NLRP3 staining. **i** Representative western blot analysis of N protein and NLRP3 in lung homogenates (*n* = 3 mice per group) at 4 dpi. All the error bars indicate standard deviations (*n* = 3). All the *P* values were calculated via one-way ANOVA. *P < 0.05; ***P < 0.001

To assess whether circSASON can be used as a therapeutic for SARS-CoV-2-induced pulmonary diseases, wild-type mice were intranasally inoculated with 10⁴ focus-forming units (FFUs) of the SARS-CoV-2 beta variant (B.1.351) to model pulmonary infection. circSASON (50 µg) or the positive control therapeutic circSASO (50 µg) was administered intranasally at 2, 24, and 72 h post-infection. Lung tissues and plasma were collected at 2 and 4 days post-infection (dpi) for analysis (Fig. 6a). Histologically, SARS-CoV-2-infected controls presented patchy parenchymal lesions with thickened alveolar septa and inflammatory cell infiltration. Notably, both the circSASON- and circSASO-treated groups exhibited attenuated pulmonary inflammation (Fig. 6b). Compared with circSASO, quantitative pathological scoring (0–5 scale) confirmed the superior protection of circSASON against SARS-CoV-2 beta variant (B.1.351)-induced pulmonary injury (Fig. 6c).

We subsequently assessed the efficacy of circSASON in reducing the lung viral load at 2 dpi, and circSASON treatment reduced pulmonary viral titers to below detection limits, outperforming both the saline controls and circSASO (Fig. 6d). Lung cytokine analysis at 4 dpi revealed significant suppression of proinflammatory cytokines (*Il6, Tnfα, Il1β, and Il18*) in circSASON-treated mice, with trivalent chimeras (circSASON) showing greater efficacy than their bivalent counterparts (circSASO) (Fig. 6e). The plasma cytokine profiles mirrored the pulmonary trends (Fig. 6f), confirming the body-level anti-inflammatory effects of circSASON. Immunohistochemical analysis revealed that robust N protein expression was diffusely localized in the bronchial and alveolar epithelia of control mice, which was significantly attenuated by circSASON treatment (Fig. 6g). Immunohistochemical statistical analysis revealed that circSASON significantly reduced N protein expression in SARS-CoV-2 beta variant (B.1.351)-infected lungs, with parallel Western blot confirmation of suppressed viral N protein expression, underscoring its superior protective efficacy against the variant (Fig. 6h, i).

Overall, these in vivo results demonstrate that circSASON can be efficiently delivered to the lung. Despite its short half-life, it exhibits low immunogenicity and exerts trivalent therapeutic effects against SARS-CoV-2 infection by suppressing viral replication (via N gene silencing) and inhibiting N protein-driven inflammatory cascades, thereby outperforming the previously generated bivalent circSASO.

## Discussion

Current antiviral therapeutic strategies predominantly target viral entry or replication but have failed to address virus-induced inflammation. In this context, we developed a novel N protein-targeting aptamer (NApt8-3) with broad-spectrum anti-inflammatory activity and employed a specific drug delivery system to validate its therapeutic efficacy in vivo. The therapeutic design strategy employed, together with the N aptamer developed in this study, may offer a framework for the rapid development of treatments to combat future pandemics caused by emerging coronavirus variants.

The coronavirus nucleocapsid (N) protein not only regulates viral genome packaging but also triggers inflammation. Its N-terminal (NTD) and C-terminal domains (CTD) shares more than 90% homology across with those of different coronaviruses, making them attractive targets for broad-spectrum drug development. However, the highly dynamic and variable structure of the N protein poses challenges for small-molecule drug discovery. In contrast, aptamers, with their high specificity, low immunogenicity, and ease of chemical modification, can effectively target the N protein, offering a promising platform for therapeutic development.^35^ In current study, we developed an aptamer, NApt8-3, that shows high-affinity binding with N proteins of broad-spectrum coronaviruses, including SARS-CoV-2 (WT, Beta B.1.351, Omicron BA.2.3), SARS-CoV, MERS-CoV, HCoV-OC43, and HCoV-229E. Compared with previously reported N protein aptamers (Seq-7, Seq-59, Seq-333, and Seq-1022),^25^ NApt8-3 exhibited significantly enhanced anti-inflammatory activity, highlighting its potential as a promising tool for developing therapeutics against a broad spectrum of coronaviruses. In this study, we further elucidated the underlying mechanism of the anti-inflammatory activity of NApt8-3. We revealed that NApt8-3 disrupts the interaction between the N protein and the NLRP3 inflammasome by binding to both the N-CTD and N-NTD. While a previous study suggested that N-CTD mediates the interaction with NLRP3,^16^ our truncation assays demonstrated that both N-CTD and N-NTD contribute to NLRP3 interaction (Supplementary Fig. 2j and k), with N-CTD displaying greater binding affinity. Importantly, molecular docking and experimental validation revealed that NApt8-3 not only engages the CTD but also directly targets positively charged arginine residues (R92, R107, and R149) within the RNA-binding NTD. These findings reveal a previously unrecognized dual-domain binding mode, highlighting NApt8-3 as a unique inhibitor capable of simultaneously interfering with both CTD- and NTD-mediated pathways of N protein–driven inflammasome activation.^36^

To date, at least 7 aptamers targeting the N protein have been developed. In 2024, Yang et al. identified six aptamers with nanomolar affinity for N proteins across multiple human coronaviruses, including SARS-CoV-2.^25^ Our study advances this field in several keyways. First, compared with Yang’s N aptamers, NApt8-3 exhibited the strongest anti-inflammatory activity in vitro (Fig. 2d–g). Second, we demonstrated that the N aptamer (NApt8-3) inhibit inflammation by blocking the interaction between N protein and the NLRP3 inflammasome, a key mediator of coronaviral-induced inflammation. Third, our N aptamer targets conserved regions on N protein, supporting its potential as a broad-spectrum lead compound against emerging coronaviruses; Fourth, none of the previously identified N aptamers have been validated as therapeutic agents in a mouse model of coronavirus-induced diseases because of the difficulty in achieving specific and efficient delivery of N aptamers into virus-infected tissues.^24,25,28,31,33^ To overcome this obstacle in aptamer delivery, we employed an S aptamer-dependent and viral infection-mediated delivery strategy to construct a trivalent aptamer chimera (circSASON) by adding NApt8-3 modules into the anti-SARS-CoV-2 chimera (circSASO) we developed previously. Compared with circSASO, circSASON had significantly greater inhibitory effects on SARS-CoV-2 replication and pulmonary inflammation in vivo. Notably, the NApt8-3 aptamer that we developed is the first and only N aptamer to date that has been functionally validated as a therapeutic agent in a mouse model of coronavirus-induced diseases.

By using the viral infection-mediated delivery strategy described in our previous paper,^31^ we developed a proof-of-concept anti–SARS-CoV-2 agent—circSASON—and demonstrated that circSASON exhibits robust anti-inflammatory activity and anti-replication activity against SARS-CoV-2 both in vitro and in vivo. The antiviral and anti-inflammatory functions of circSASON outperformed our previously generated bivalent circSASO in a mouse model, indicating that circSASON is a lead compound with great therapeutic potential. In line with prior studies showing that aptamer exbibit low immunogenicity, intranasal administration of circSASON did not significantly induced expression of cytokines (IL-6, TNF-a and IFN-g) in both lung and plasma, highlighting a favorable safty profile. However, consistent with previous research,^21,37,38^ our results showed that these unmodified aptamers (circSASON) exhibit relatively poor metabolic stability and rapid renal clearance. Following intranasal administration, circSASON was detected in liver, lung, kidney and spleen at 2 h, with the highest levels in the lung and liver. At 48 h, circSASON levels markedly decreased in the lung and kidney, while accumulation increased in the liver, suggesting organ-specific clearance and metabolism patterns. These findings demonstrated circSASON, as an unmodified aptamer, it exhibits relatively poor metabolic stability and rapid clearance, which may limit its in vivo persistence and clinical translation. Thus, future work on its chemical modifications—such as 2′-O-methylation, PEGylation, or incorporation of locked nucleic acids—to enhance the pharmacokinetic profile, metabolic stability, and systemic persistence is needed. Importantly, assessing absorption, distribution, metabolism, and excretion (ADME) properties is essential for translating circSASON into a potential clinical candidate (PCC). Given that circSASON has already demonstrated therapeutic potential in preclinical models, it represents a promising lead compound for future drug development.

Despite the official end of the COVID-19 pandemic, several significant unresolved challenges remain: (1) the persistent presence of SARS-CoV-2 in the human body, which contributes to chronic inflammation and long-term organ damage associated with long COVID-19^7,8^; (2) the ongoing mutation and emergence of new coronavirus variants pose a continuing threat to global health.^39,40^ These factors underscore the critical need for innovative therapeutic strategies that can both limit viral replication and alleviate virus-induced inflammation. The development of effective therapeutics against coronavirus remains an ongoing priority, particular those capable of rapid adaptation to emerging variants. Our findings highlight the potential of the N aptamer (NApt8-3) to mitigate coronavirus-induced pulmonary inflammation, demonstrating robust anti-inflammatory activity while maintaining a favorable safety profile. By targeting key pathogenic mechanisms, NApt8-3 offers a promising strategy not only for the treatment of acute coronavirus infections but also for reducing the risk of long-term sequelae, providing a versatile platform for the rapid development of therapeutics to combat future pandemics caused by emerging variant coronavirus outbreaks.

## Materials and methods

### Materials

His-tagged Nucleocapsid recombinant protein of SARS-CoV-2(WT) was obtained from ACRO Biosystems Inc. (NUN-C5227, USA). His-tagged Spike trimer recombinant protein of SARS-CoV-2 was purchased from Novoprotein Scientific Inc. (DRA49, Shanghai, China). SARS-CoV-2 Spike (WT) Fluc-GFP Pseudovirus was purchased from ACRO Biosystems Inc. (PSSW-HLGB001, USA). SARS-CoV-2 Nucleocapsid Phosphoprotein Rabbit Polyclonal antibody was purchased from Proteintech Inc. (28769-1-AP, USA). SARS-CoV/SARS-CoV-2 Nucleocapsid Mouse Polyclonal antibody (40143-MM05), SARS-CoV-2 Nucleocapsid NTD protein (40588-V07E10), SARS-CoV-2 Nucleocapsid CTD protein (40588-V07E5) were purchased from Sino Biological Inc. (Beijing, China). NLRP3 protein (ab165022) was purchased from abcam (Cambridge, UK). Anti-NLRP3 rabbit polyclonal antibody (D120143), Anti-ASC rabbit polyclonal antibody (D154049) was purchased from Sangon Biotech (China). HRP-conjugated Beta Actin Monoclonal antibody was purchased from Proteintech Inc. (HRP60008, USA). His-tag protein pure Ni-Beads (Ni-Beads) were purchased from BioMag Beads (BMNI-5, Wuxi, China). Polyinosinic-polycytidylic acid (Poly(I:C)) and Resatorvid were obtained from MedChemExpress (HY-107202/HY-11109, Shanghai, China). Dip and Read^TM^ Biosensors streptavidin (SA) were purchased from ForteBio (California, USA). All media for cell culture were purchased from Gibco (USA). Fetal bovine serum (FBS) was purchased from Excell Bio (Shanghai, China) and penicillin-streptomycin was purchased from Hyclone (USA). All types of DNA sequences with HPLC purification were synthesized by Sangon Biotech (Shanghai, China).

### Cell culture

The hACE2-HEK293T cells were gifts from Prof. Zhao Jincun at the Guangzhou Institute of Respiratory Disease. Calu-3 cells (CL-0054) were obtained from Procell Life Science& Technology Co., Ltd (ATCC, HTB-55 BCRJ, 0264).hACE2-HEK293T cells were cultured in DMEM medium (Gibco) supplemented with 100 U/mL Penicillin-Streptomycin, 2 μg/mL Puromycin and 10% (v/v) fetal bovine serum (ExCell Bio), in a humidified atmosphere containing 5% CO_2_ incubator at 37 °C. Calu-3 cells were cultured in MEM medium (Gibco) supplemented with 100 U/mL Penicillin-Streptomycin and 10% (v/v) fetal bovine serum (ExCell Bio), in a humidified atmosphere containing 5% CO_2_ incubator at 37 °C. Cells were negative for mycoplasma.

### Micro-well SELEX procedures

His-tagged full-length SARS-CoV-2 Nucleocapsid protein was used as the bait and His-tag protein was used as the negative control. A synthetic ssDNA library (5′-TGCGTGTGTAGTGTGTCTG-[N]_40_-CTCTTAGGGATTTGGGCGG-3′ (*N* = A, T, G, or C)) consisting of 40 nt random core sequence and constant 19 nt arm sequences at both ends were used for target-based SELEX. Briefly, the synthetic ssDNA library was incubated with His-tagged Nucleocapsid proteins conjugated to LOCKWELL C8 MAXISORP (Microplate, 446469, Thermo Fisher Scientific, USA) for SELEX enrichment. This Microplate conjugated His-tagged nucleocapsid proteins was incubated with the library at 25 °C for 30 min in the binding buffer contained 1×PBS buffer with magnesium ions (10 mM Na_2_HPO_4_, 2 mM KH_2_PO_4_, 137 mM NaCl, 2.7 mM KCl, 0.55 mM MgCl_2_, pH = 7.4), and then separated and washed twice with the washing buffer (PBST: the binding buffer with 0.05% Tween 20). Subsequently, the bound oligonucleotides were eluted by Milli-Q H_2_O at 80 °C for 7 min with mild shaking. The selected ssDNA was amplified by PCR. To convert dsDNA to ssDNA, the Lambda exonuclease enzyme was applied to separate and remove the 5′-phosphorylated antisense sequences from the sense sequences. Then, the ssDNA purified using NucleoSpin® Extract II kit was subjected for the next round of SELEX. After 6 rounds, the enriched ssDNA sequences were subjected to counter-selection with the total protein in human lung epithelial cells (A549). After 12 rounds of selection, the enriched aptamer pools were assessed by high-throughput sequencing (Sangon Biotech, Shanghai, China), and the most abundant ssDNA were selected as candidate N protein aptamers.

### Enzyme linked immunosorbent assay (ELISA)

Microplates were pre-coated with all kinds of Nucleocapsid proteins (8 nM) in 100 μL coating buffer (pH = 9.6, 100 mM NaHCO_3_) at 4 °C overnight. After washing two times, 200 μL blocking solution (2% BSA in PBST) was added at room temperature (RT) for 1 h with mild shaking. After blocking, serial dilutions of biotin-labeled aptamers at the 5′-end were added into microplates and incubated at RT for 30 min. Meanwhile, the blocking solution and random sequences were used as baseline control, respectively. Microplates were washed twice to remove free aptamers. Next, streptavidin-horseradish peroxidase (strep-HRP, 1:2000, Solarbio, SE068) was sequentially added into the reactions. After washing three times, the 3,3′,5,5′-Tetramethylbenzidine (TMB) substrate was added to the microplates (100 μL per well) and incubated for 15 min at RT. The reaction was stopped by the addition of 2 M H_2_SO_4_ (50 μL per well) and the absorbance was measured at 450 nm using a microplate reader.

### Flow cytometry analysis

To evaluate the binding performance of selected aptamers, His-tagged SARS-CoV-2 Nucleocapsid proteins (40 nM) coated in Ni-beads were incubated with 200 nM cy3-labeled aptamers in 100 μL binding buffer at RT for 30 min. Meanwhile, Ni-beads incubated with cy3-labeled random sequences were used as the negative control. The beads were washed twice using washing buffer and suspended in 1 mL binding buffer. The fluorescence intensity of beads with counting about 5000 events was measured by flow cytometry2 (FACSVerse, BD). Meanwhile, the data was analyzed using FlowJo (V10, BD). Cells were harvested 48 h after transfection and resuspended in 1 × PBS with 4% FBS and kept on ice before flow cytometry analysis. Flow cytometry data was collected using BD FACScalibur (BD, USA) and analyzed in two channels: green (excitation with 488 nm, emission with 525 ± 25 nm) or red (excitation with 561 nm, excitation with 610 ± 10 nm). The data is processed and analyzed in the FlowJo software.

### Fluorescence microscope imaging

Ni-beads with His-tagged SARS-CoV-2 Nucleocapsid protein (5 μL beads and about 2 μg Nucleocapsid protein) or negative Ni-beads with His-tagged protein were incubated with 200 nM FAM-labeled aptamers in 100 μL binding buffer at RT for 30 min. After washing two times using washing buffer, the beads were suspended in 100 μL binding buffer and the fluorescence was monitored by fluorescence microscope (Leica DMi 8, USA). The fluorescence intensity was quantified by Image J software (2021, USA).

### Bio-layer interferometry (BLI)

BLI was carried out according to our previous research.^41^ Briefly, the sensors were immersed into 96-well added 200 μL PBST/Mg^2+^ (0.55 mM) and shaking (1000 rpm) for 90 s (baseline phase). For loading purposes, streptavidin biosensors were used to capture biotinylated DNA (the thickness signal was 0.6 nm) in PBST/Mg with shaking for 10 min (loading phase). After washing in the PBST/Mg^2+^ for 60 s (second baseline phase), the loaded sensors were immersed into a serial dilution of S proteins with shaking for 3 min (association phase). Then, the sensors were immersed into PBST/Mg^2+^ for an additional 3 min (disassociation phase). Binding affinity kinetic features using ForteBio Octet K2 (Pall, USA).

### Molecular docking and dynamic simulations

The structures of N proteins from SARS-CoV-2, SARS-CoV, MERS-CoV, HCoV-OC43, HCoV-229E, and NLRP3 were retrieved from the RCSB Protein Data Bank (http://www.rcsb.org). Molecular docking was carried out according to our previous research.^26,41^ Briefly, Molecular docking was performed with HDOCK after obtaining the structures of aptamers and their target. The complex structure between the protein and aptamer was predicted using a hybrid protein-DNA docking algorithm, HDOCK. HDOCK used the fast Fourier transform (FFT)-based search strategy to globally sample all possible binding modes between the two molecules. All the sampled binding modes were evaluated by the iterative knowledge-based scoring function ITScorePP. Ultimately the binding modes were ranked according to their binding energy scores, and the top binding modes were provided. During the docking calculation, all the default parameters were used. The binding residues analysis was performed by the HDOCK webserver.

### Co-IP

A549 cells were transfected with N-Flag plasmid and NApt8 or NApt8-3 for 48 h, meanwhile, cells were stimulated with LPS. After processing, cells were harvested and resuspended in 1 × PBS with 0.1% NP-40. Cells were centrifuged at 12,000 × *g* after ultrasonic disruption and harvested the supernatant. Flag-beads were added to the supernatant for mild shake overnight. The Flag-beads were used for SDS-PAGE after washed three times by 1 × PBS with 0.1% NP-40. Then, the gels were for silver staining analysis, which differential bands will be analyzed by Mass Spectrometry.

### Western blot analysis

The hACE2-HEK293T cells and Calu-3 cells were washed twice with PBS and dissolved in lysis buffer (50 mM Tris-HCl pH 7.4, 300 mM NaCl, 1% Triton X-100, 5 mM EDTA, 100 mM phenylmethylsulfonyl fluoride (PMSF) and 10% glycerol) after transfection using N with or without aptamer and chimera. Protein concentration was measured by Bicinchoninic Acid assay (BCA). Proteins were separated by SDS polyacrylamide gel electrophoresis (SDS-PAGE), transferred onto PVDF membranes (Bio-Rad) and incubated overnight at 4 °C with corresponding antibodies: anti-N (1:2000), anti-NLRP3(1:2000), anti-ASC (1:1000), anti-GAPDH (1:20000), anti-ACTB-HRP (1:10000). A HRP conjugated Goat anti-mouse IgG or Goat anti-rabbit IgG secondary antibody (1:10000) was used for the detection of primary antibody. The protein signals were detected using ECL chemiluminescent substrate (FOREGENE, China).

### Confocal microscope of hACE2-HEK293T cells treated with SARS-CoV-2 pseudovirus

Cy3-labeled DNA (200 nM) was incubated with SARS-CoV-2 pseudovirus (low: 1 × 10^4^ TCID_50_/mL, high: 1 × 10^5^ TCID_50_/mL, 1 TCID_50_ ≈ 0.7 PFU) in binding buffer at RT for 10 min. Then, 1 × 10^5^ HEK293T-ACE2 cells were incubated with mixtures of SARS-CoV-2 pseudovirus and DNA at 37 °C for 48 h. After incubating with Hoechst 33342 for 15 min, hACE2-HEK293T cells were observed to image with a Zeiss CELL Observer SD confocal microscope with a 60 × oil objective. The excitation wavelengths were 488 nm (Green Channel) and 561 nm (Red Channel). Exposure times: 200–400 ms. Acquired images were analyzed by the ZEN (blue edition).

### Assessment of inflammatory responses of cells

Calu-3 or A549 cells were co-transfected with pan-coronavirus N plasmids and NApt8-3 or NASO2 with Lipofectamine 2000 following the manufacturer’s protocol. After 48 h, the cell was harvested. Subsequently, Trizol was used to extract RNA. qRT-PCR was used to quantitate the mRNA levels of inflammatory cytokines, including IL-6, TNFa, IFN-g.

The SARS-CoV-2 strains used in this analysis were isolated from COVID-19 patients in Guangdong, China, including wild type (SARS-CoV-2/human/CHN/IQTC-01/2020, NCBI, accession number: MT123290), Beta (B.1.351), Omicron (BA.2.3). Authentic SARS-CoV-2 virus (2 × 10^6^ PFU/mL, MOI = 0.01) were mixed with circular chimera in binding buffer at RT for 10 min. Then, 5 × 10^5^ of Calu-3 cells were incubated for 48 h. After incubating, cells were harvested to detect the inflammatory cytokines. All authentic SARS-CoV-2 infection experiments were performed in Guangzhou Gustoms District Technology Center Biosafety Level 3 (BSL-3) Laboratory.

### Agarose gel electrophoresis analysis of circular chimera stability

Three μg chimera/circular chimera was added to the binding buffer containing 10% FBS and incubated at 37 °C for different times (0, 6, 12, 24, 36, 48 and 72 h). Then, the SN/circSN, SASON/circSASON and Random sequences were loaded onto 2% agarose gel in 1×TAE buffer and run at 120 V for 30 min. Gels were incubated in Ethidium bromide (1 μg/ml) for 5 min and bands were imaged using Gel Doc XR + Gel imaging system (Bio-rad, Hercules, California, USA) with ultraviolet excitation (302 nm). The fluorescence intensity was quantified by Image J software (2022, USA).

### Immunofluorescence assay (IFA)

A series of circSASON and control oligonucleotides (SApt, RS etc) were incubated with authentic SARS-CoV-2 virus (2 × 10^6^ PFU/mL, MOI = 0.01) at 37 °C for 1 h. Then, 1 × 10^5^ Vero E6 cells were incubated with mixtures of SARS-CoV-2 virus and oligonucleotides at 37 °C for 48 h or 72 h. For immunofluorescence assay, Vero E6 cells were incubated in Paraformaldehyde at 4 °C overnight for permeabilization. The expression of nucleocapsid protein from the SARS-CoV2 virus was detected by using N protein primary antibody for 1 h at 37 °C. After washing with PBST, wells were incubated with secondary antibody for 1 h at room temperature. Meanwhile, the cell nucleus was stained with DAPI. Images were acquired with the fluorescence microscope and analyzed with Image J software.

### Animal model

Female BALB/c mice (4–5 weeks old) were purchased from Guangzhou GemPharmatech Co. Ltd or Chengdu GemPharmatech Co. Ltd. All the mice were kept in sterile, autoclaved cages and provided with enough food and water. All animal experiments were undertaken at Sichuan University and Guangzhou Medical University. All protocols were approved by the Institutional Animal Care and Use Committees of the Sichuan University and of the Guangzhou Medical University.

Female BALB/c mice (4–5 weeks old) were purchased from Guangzhou GemPharmatech Co., Ltd. All animal experiments using SARS-Co-2 were undertaken at Guangzhou Medical University and approved by the local regulatory agency. SARS-CoV-2 Beta B.1.351 (2000 FFU) were mixed with saline for 1 h. Then, mice were administrated intranasally with the mixture. At 2 h, 24 h and 72 h after viral infection, circSASON or circSASO (2.5 mg/Kg body weight) were administrated intranasally. Lung and blood samples was obtained at 2 dpi and 4 dpi. Lungs were used for virus quantification using focus forming assay and used for pathological analysis using H.E. staining or Immunohistochemistry staining. Plasma was used for evaluation of inflammation using qRT-PCR and ELISA. All animal experiments were performed with a minimal of 3 mice per experimental condition.

### Focus forming assay (FFA)

FFA was performed as previously described.^32^ Compared with the traditional plaque assay, FFA provides higher throughput. Vero E6 cells were seeded in 96-well plates one day prior to infection. Serially diluted virus stocks or lung homogenate were added to the Vero E6 cells and inoculated at 37 °C for 1 h. After incubation, the inocula were removed and 125 μL of pre-warmed 1.6% carboxymethylcellulose was added to each well. At the indicated time points post-infection, cells were harvested, and foci were visualized using a peroxidase substrate and counted under a microscope. Viral titers were calculated as focus-forming units (FFU) per milliliter.

### Histology and IHC

Lung tissues were harvested and immersion-fixed in 4% paraformaldehyde overnight. Paraffin sections were prepared and stained. Antibodies targeting the SARS-CoV-2 nucleoprotein (N) (Sino Scientific, no. 40143-R019, 1:1000) and NLRP3 (Adipogen, no. AG-20B-0014-C100, 1:200) were used. Pathology evaluation were performed as described in the previous research,^42,43^ injuries such as bleeding and edema of the entire left lung tissue can be observed by H&E staining under low microscope magnification (10×), the evaluation method is to observe injury distribution from 6 independent visual fields and score them following criteria (injury area/total area):

0 points: no damage;

- 1 point: mild injury, <25% mild influx of inflammatory cells with cuffing around vessels;
- 2 points: moderate injury, increased inflammation with ~25–50% of the total lung involved;
- 3 points: severe inflammation involving 50–75% of the lung;
- 4 points: almost all lung tissue contained inflammatory infiltrates.

### Quantitative RT-PCR (qRT-PCR) analysis

Total RNA was isolated from the stimulated cells by RNAiso Plus (108-95-2, TaKaRa, Japan). Then, cDNA was prepared using DNase I (01056834, Thermo Scientific, USA) and RevertAid Reverse Transcriptase (00991337, Thermo Scientific, USA). qRT-PCR was performed using the Applied Biosystems 7500 Real-Time PCR Systems (Thermo Fisher Scientific, USA) with 2×Real PCR EasyTM Mix-SYBR (210520, Foregene, China). ACTB was used as a reference gene. The quantitative analysis of qRT-PCR to evaluate the inhibitory efficiency of aptamer on cytokine gene expression were analyzed by the Livak method (2^-ΔΔCt^), described as following steps:

1. $$\Delta \mathrm{Ct\; calculation}:\Delta \mathrm{Ct}=\mathrm{Ct}(\mathrm{mRNA})-\mathrm{Ct}(\mathrm{actin})$$
2. $$\Delta \Delta \mathrm{Ct\; calculation}:\Delta \Delta \mathrm{Ct}=\Delta \mathrm{Ct}(\mathrm{sample})-\Delta \mathrm{Ct}(\mathrm{blank})$$
3. $$\mathrm{Fold\; change}:\mathrm{Fold}={2}^{(-\Delta \Delta \mathrm{Ct})}$$
4. $$\mathrm{Relative}\,\mathrm{inhibition}({\rm{ \% }})=100-(\mathrm{Fold}(\mathrm{sample})/\mathrm{Fold}({\rm{N}}))\times 100$$

### Bliss independence analysis

To calculate Bliss independence for evaluating drug synergy, you compare the expected combined effect of two drugs (assuming independence) with the observed effect of their combination. Bliss Independence Formula: *E*_*A*_ = effect of drug A alone. *E*_*B*_ = effect of drug B alone. *E*_*AB*_ = observed effect of the combination. The expected effect under Bliss independence is: *E*_Bliss _= *E*_*A*_ + *E*_*B*_ − *E*_*A*_⋅*E*_*B*_. If observed *E*_*AB*_>*E*_Bliss_, it is considered that the combination of A and B exerts a synergistic effect.

### Statistical analysis

All analyses were repeated at least three times, and a representative experimental result was presented. Data were analyzed using GraphPad Prism version 8.0 (GraphPad Software, San Diego, CA). Continuous variables with normal distribution are expressed as the mean ± standard deviation (SD). Comparisons between groups were all verified for normal distribution by D’Agostino-Pearson omnibus test. Student’s *t* test (for pairwise comparisons) and one-way ANOVA (for comparisons among three or more groups) were used. The post hoc test with Bonferroni correction was performed for multiple comparisons following ANOVA.

## Supplementary information

Targeting coronaviral inflammation: aptamer-based strategies for emerging threats

dCT value

## Data Availability

SELEX sequencing data have been deposited in the NCBI Sequence Read Archive database under accession numbers PRJNA1390424. The mass spectrometry data are available via ProteomeXchange with identifier PXD072190. All other data generated in this study are included in the published article and its supplementary data files.
