# Supplementary material for: Targeting coronaviral inflammation: aptamer-based strategies for emerging threats
Source: Signal Transduct Target Ther. 2026 Feb 13;11:54. doi: 10.1038/s41392-025-02570-8 (PMC12901053; doi:10.1038/s41392-025-02570-8)
Supplement: Supplementary file 1 — Targeting coronaviral inflammation: aptamer-based strategies for emerging threats [file 41392_2025_2570_MOESM1_ESM.docx]

Supplementary Materials for

Targeting coronaviral inflammation: aptamer-based strategies for emerging threats

Yongyun Zhao^1, 2, †^, Gang Yang^1, 2, †^, Zhaoyong Zhang^3, †^, Mingfeng Xie^1, 2, †^, Junnan Liu^1^, Yiran Cheng^1, 2^, Yabin Zhang^1^, Xinyu Zhang^1^, Yuchun Wang^1^, Duhan Ma^1, 2^, Longteng Tang^1, 2^, Wei Li^1, 2^, Yanxin Huang^2^, Yongli Bao^2^, Jincun Zhao^3^, Xu Song^1,^ *, Fengming Luo^1,^ *, Huajing Wan^1,^ *

Correspondence to: [wanhuajing1974@wchscu.cn](mailto:wanhuajing1974@wchscu.cn), [fengmingluo@outlook.com](mailto:fengmingluo@outlook.com), [xusong@scu.edu.cn](mailto:xusong@scu.edu.cn)

**This PDF file includes:**

Materials and Methods

Figures. S1 to S6

Tables S1 to S2


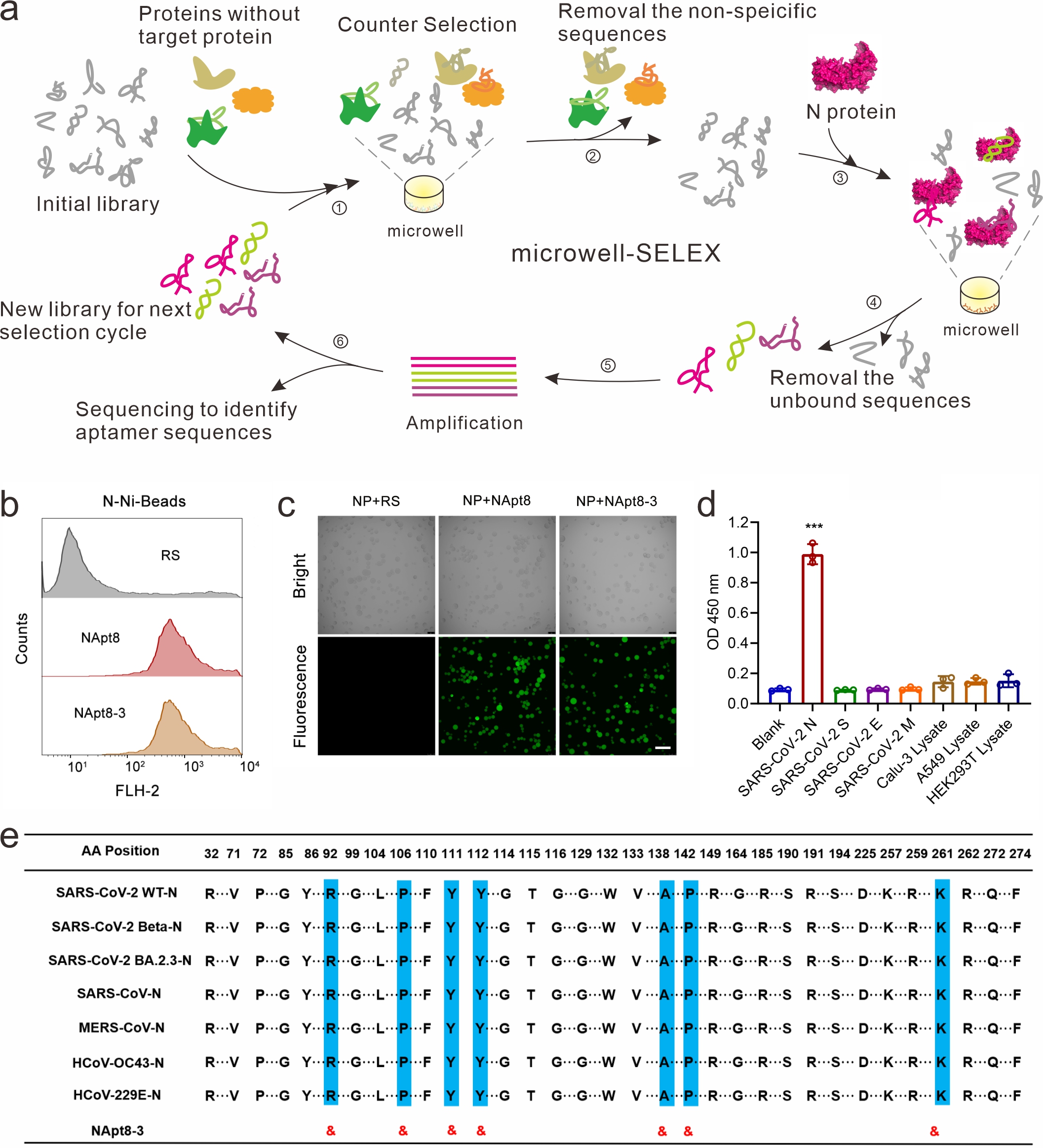


**Figure. S1**. **a.** The scheme of microwell-SELEX that coated proteins on plates for the discovery of aptamers against the N protein. **b**. Flow cytometry to investigate the binding performance of cy3-labeled aptamer (200 nM) to N protein (2 μg) in cell lysate. Random sequences (RS) were used as baseline controls. **c**. Fluorescence images to detect the binding performance of FAM-labeled candidate sequences (200 nM) to 100 μL binding buffer containing His-NP-beads (5 μl beads and 2 μg His-N protein. **d.** Off-target binding analysis of NApt8-3 against proteins of SARS-CoV2 and HEK293T cell lysate. **e**. The conserved binding sites of NApt8-3 to N protein of virous coronavirus. All the error bars indicate standard deviations (n=3). The *P* values were calculated using one-way ANOVA. ****P*＜0.001.


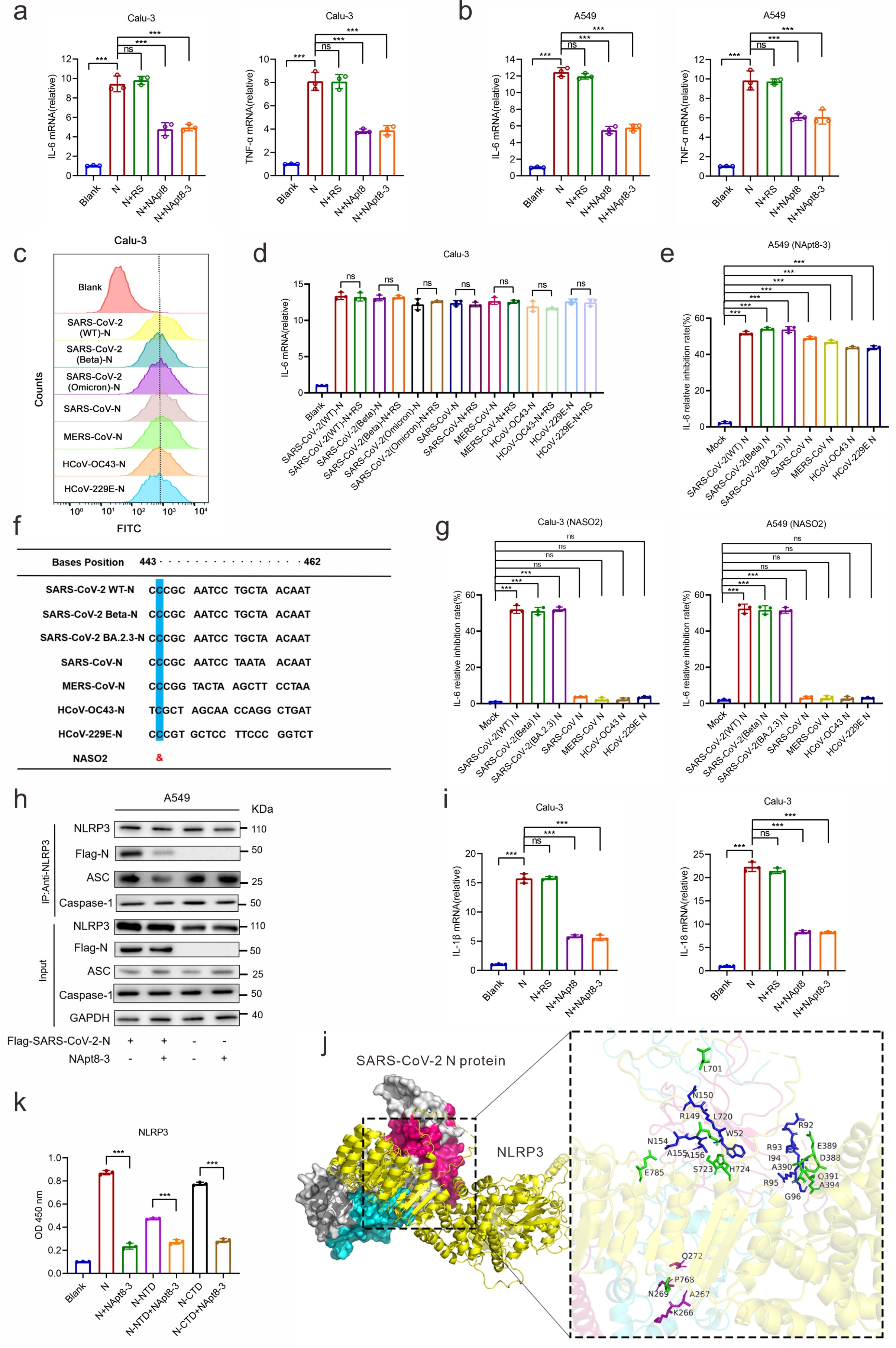


Figure. S2. a-b. qRT-PCR analysis of IL-6, TNF-α in Calu-3 and A549 cells transfected with different aptamers. The concentration of RS, NApt8, NApt8-3 was 100 nM. Blank: the cells without any treatment. mRNA (relative) indicates the mRNA expression level normalized to that of actin mRNA. c. Flow cytometry analysis of N-protein expression in cells transfected with the N plasmid (1.2 μg/12 well). d. The level of IL-6 induction by each N protein alone and co-transfection with random sequence (RS). The amount of N plasmid transfected was 1.2 μg; the co-transfected amounts of N plasmid and RS were 1.2 μg and 200 nM, respectively. e. qRT-PCR analysis of IL-6 in Calu-3 cells co-transfected with the Napt8-3 and plasmids expressing N protein of virous coronavirus (SARS-CoV-2 and variants, SARS, MERS, HCoV-OC43, HCoV-229E). Mock: the cells transfected with N-encoding plasmid without DNA treatment. f. The binding site of NASO2 that silences the N gene of SARS-CoV-2 and variant. g. qRT-PCR analysis for the expression of cytokines (IL-6) in Calu-3 and A549 cells co-transfected with the NASO2 and plasmids expressing N protein of virous coronavirus (SARS-CoV-2 and variants, SARS, MERS, HCoV-OC43, HCoV-229E). Mock: the cells transfected with N-encoding plasmid without DNA treatment. h. Co-immunoprecipitation with anti-NLRP3 antibodies to confirm the interaction of N protein and NLRP-3 in the lysates of A549 cells with the indicated treatment. Cell lysates (40 μg) were used as Input. i. qRT-PCR analysis for the expression of cytokines (IL-1β and IL-18) in Calu-3 cell with the indicated treatment. Blank: the cells without any treatment. j. Molecular docking analysis of NLRP3- N protein complex. Magenta and cyan represent NTD and CTD, respectively. NLRP3 is shown in yellow. The N protein sites that interact with NLRP3 are shown with residue names. k. Competition ELISA to evaluate the efficiency of the NApt8-3 (200 nM) that prevent the indicated protein (200 ng/well) binding to NLRP3 (100 ng/well) pre-coated on microplates. NLRP3 interacts with N, NTD (NTD domain of N protein) and CTD (domain of N protein) and these interactions can all be inhibited by NApt8-3. All the error bars indicate standard deviations (n=3). The *P* values were calculated using one-way ANOVA. ****P*＜0.001.


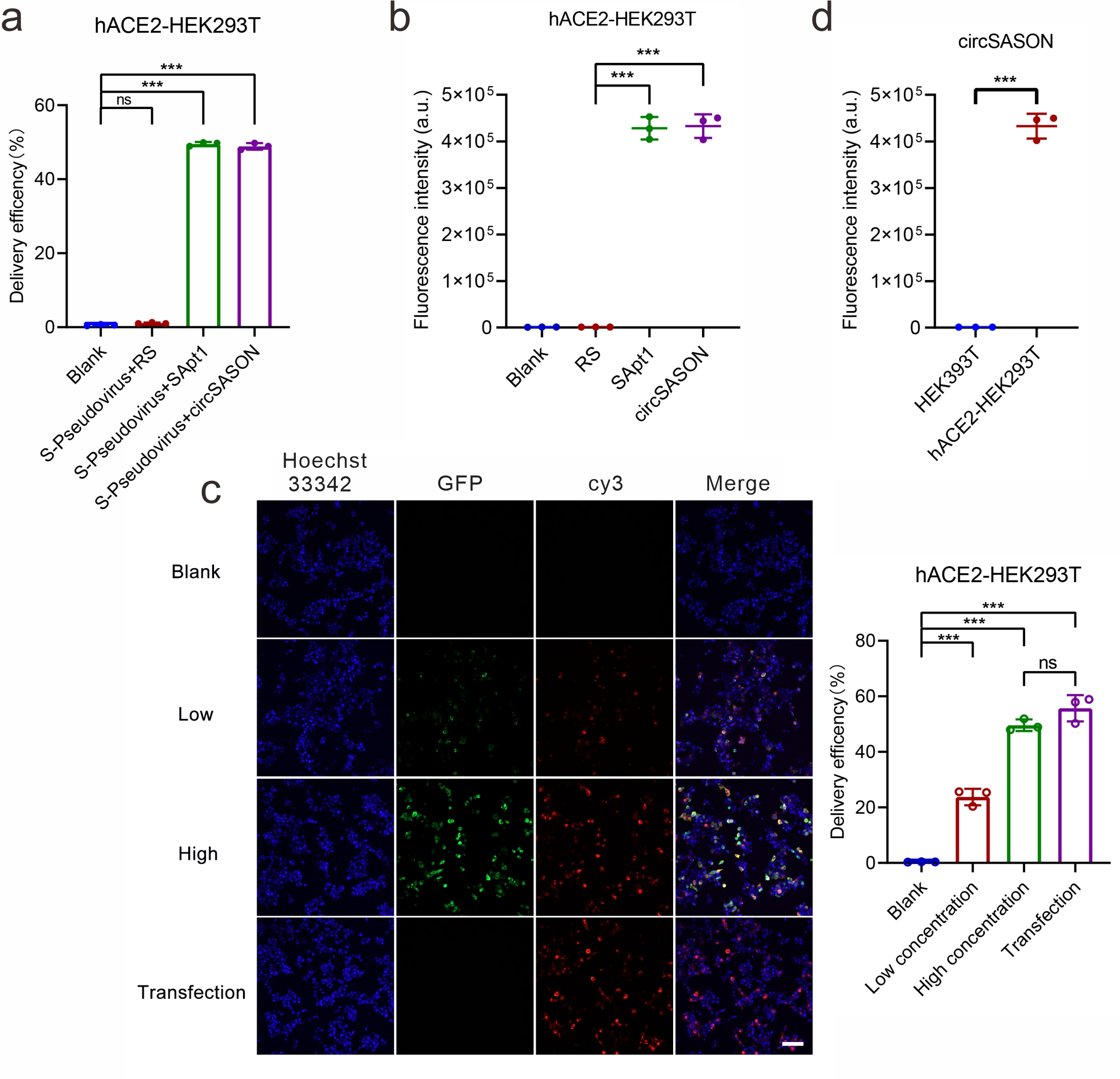


Figure. S3. a. Flow cytometry quantitative analysis of SApt-dependent and SARS-CoV-2 infection-mediate delivery of the circSASON into hACE2-HEK293T cells with treatment as indicated. b. The quantification results of Fig. 3c, in the main text. c. Viral dose-dependent delivery of circSASON chimera into hACE2-HEK293T cells. Representative fluorescence confocal imaging and quantitative analysis of hACE2-HEK293T cells incubated with the indicated SARS-CoV-2 Spike (WT) Fluc-GFP pseudovirus (Low: 1 × 10^4^ TCID50 mL^-1^; High: 1 × 10^5^ TCID50 mL^-1^), and cy3-labeled circSASON chimera (200 nm) for 48 h. Transfection: cells transfected with cy3-labeled circSASON using lipo2000. Scale bars represent 50 µm. d. The quantification results of Fig. 3d, in the main text. All the error bars indicate standard deviations (n=3). a-c: The *P* values were calculated using one-way ANOVA. d: The *P* values were calculated using t-test. ****P*＜0.001.


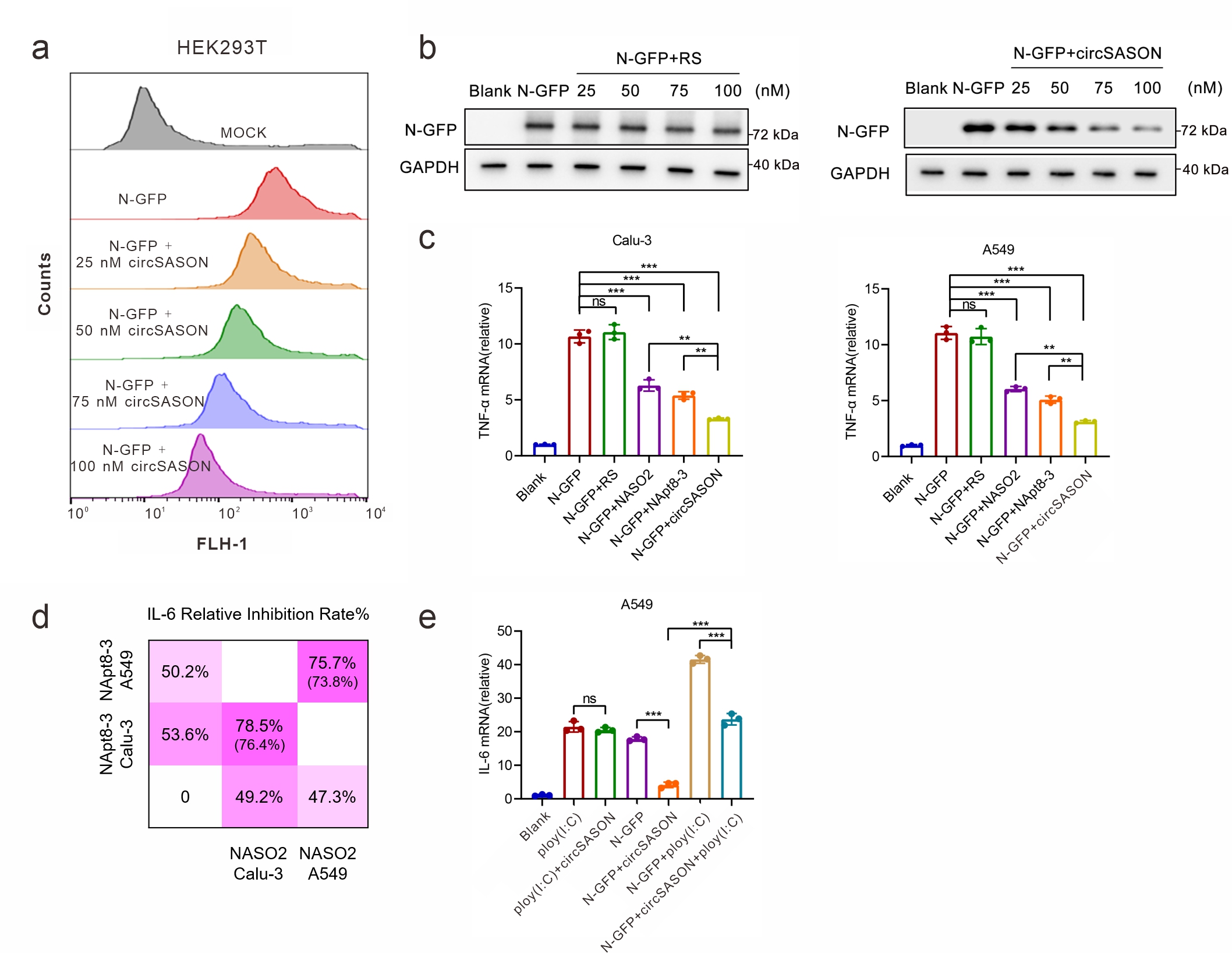


Figure. S4. a. Flow cytometry analysis of the bright fluorescent cell population with the indicated treatment. b. Representative western blot analysis of the N protein in cells treated with circSASON in dose-dependence. c. qRT-PCR analysis for the expression of TNF-α in Calu-3 cells and A549 cells with treatment as indicated. The *P* values were calculated using one-way ANOVA. d. Bliss independence analysis the data of the main Fig. 4 h. The value in parentheses represents *E_Bliss_*. *E_Bliss​_*=*E_NASO2_*​+*E_NApt8-3_*​−*E_NAOS2_*​⋅*E_NApt8-3_*_​._  *E_circSASON_*_​_>*E_Bliss_*_._ The value of E*_Bliss_* is given in parentheses. The value above the parentheses is *E_circSASON_*_​._ e. qRT-PCR analysis of immune response to Poly (I: C) in Calu-3 cells with the indicated treatment. The last sample was A549 cells pre-co-transfected with a plasmid encoding the N-GFP protein and circSASON (100 nM). Cells were then transfected with Poly (I: C) (5 µg mL^-1^) to induce cytokine again. All the error bars indicate standard deviations (n=3). The *P* values were calculated using one way ANOVA. ****P*＜0.001.

**
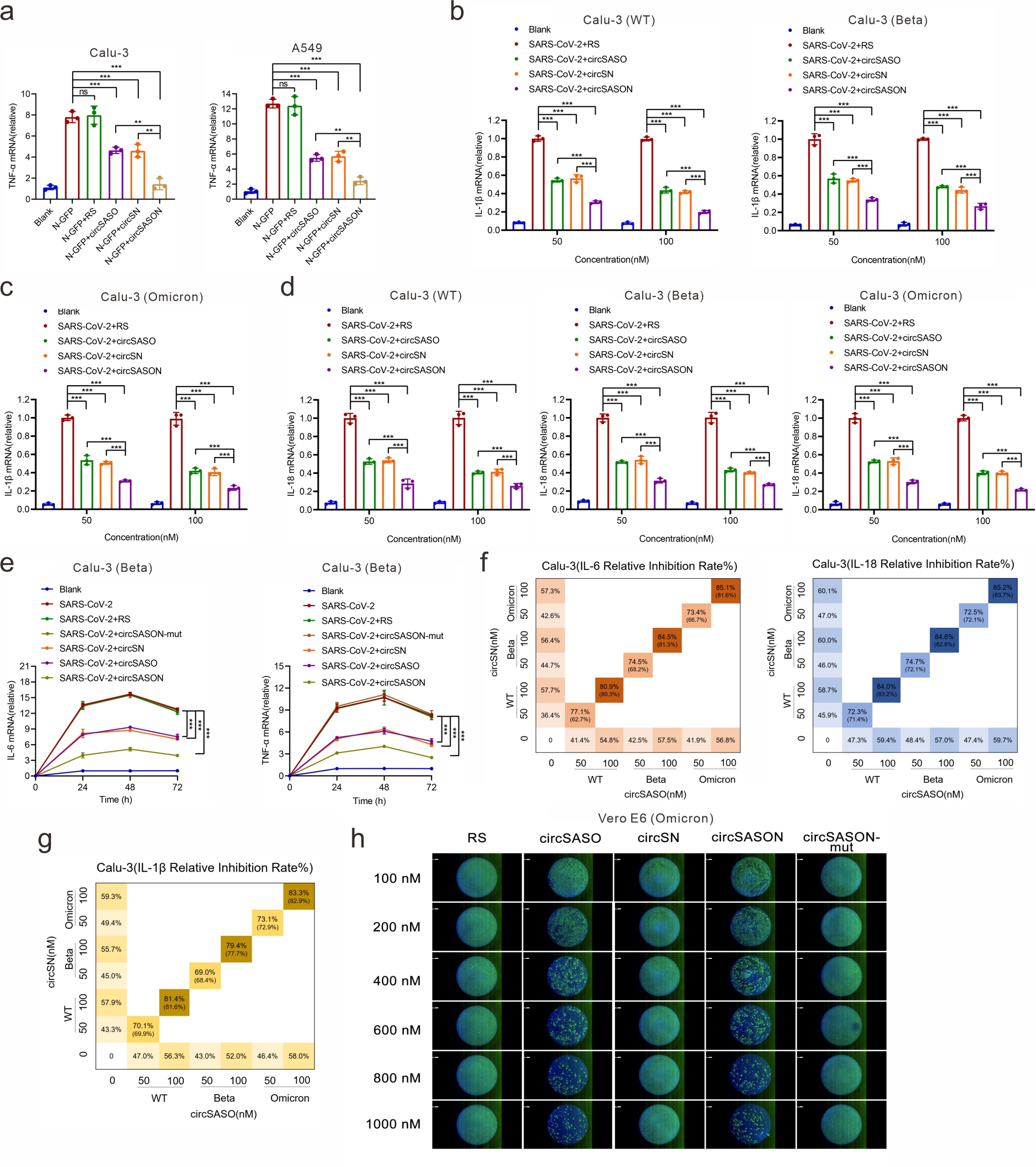
**

Figure. S5. a. qRT-PCR analysis for the expression of TNF-α in Calu-3 cells and A549 cells with treatment as indicated. b-d. qRT-PCR analysis of the inhibitory of three circular chimeras on cytokine production in cells infected with authentic SARS-CoV-2 or variants (Beta and Omicron BA.2.3). e. A time-course experiment with Calu-3 cells monitor anti-N-induced inflammatory under treatment with circSASO, circSN, and circSASON over time. f-g. Bliss independence analysis the data of the main Fig. 5c-d. *E_Bliss_*_​_=*E_circSASO_*​+*E_circSN_*​−*E_circSASO_*​⋅*E_circSN_*​_._  *E_circSASON_*_​_>*E*_Bliss._ The value in parentheses represents *E_Bliss_*. The value above the parentheses is *E_circSASON_*_​._ h. Immunofluorescent staining assay of Beta and Omicron BA.2.3 replication in Vero E6 cells incubated with authentic Omicron BA.2.3 and three circular chimeras for 48 h. Scale bars represent 1 mm. All the error bars indicate standard deviations (n=3). a, e: the *p* values were calculated using one-way ANOVA. b-d: the *P* values were calculated using two-way ANOVA. ****P*＜0.001.

**
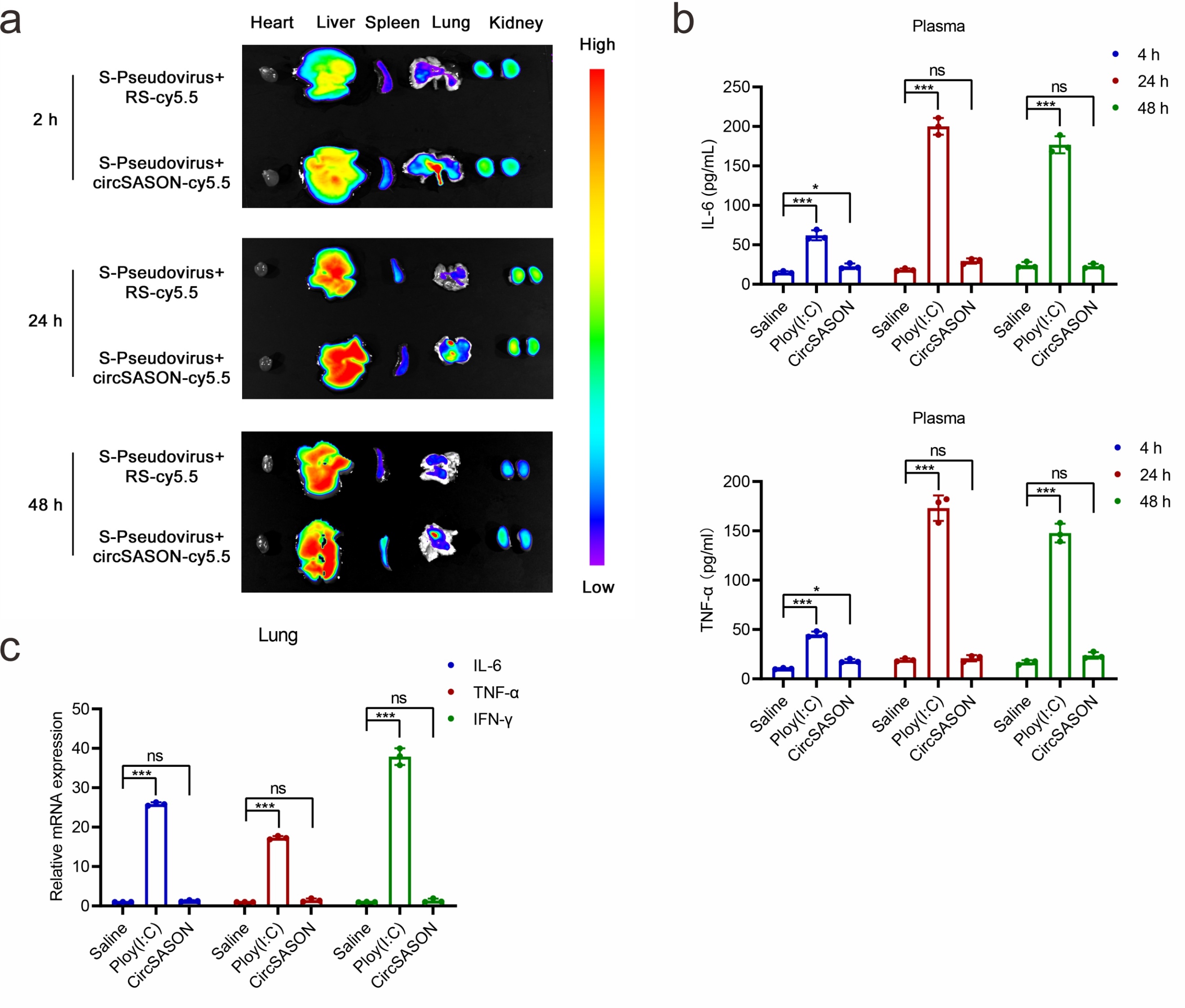
**

**Figure. S6**. **a**. Biodistribution of the chimera in mice following intranasal administration was assessed at the indicated time points. All oligonucleotides (1 nmol) were Cy5.5-labeled and mixed with SARS-CoV-2 Spike (WT) Fluc-GFP pseudovirus (1 × 10⁵ TCID₅₀/ml). **b.** Plasma cytokine levels (IL-6, TNF-α) were quantified by ELISA at the indicated times after intranasal treatment with 20 mg/kg circSASON or 5 mg/kg poly (I:C) (n = 3 mice per group). **c**. Lung tissues were harvested 48 h after intranasal treatment with 20 mg/kg circSASON or 5 mg/kg poly(I:C). mRNA levels of IL-6, TNF-α and IFN-γ were determined by qRT-PCR (n = 3 mice per group). All the error bars indicate standard deviations (*n* = 3). All the *P* values were calculated using two-way ANOVA. ****P*＜0.001.

Table S1. Mass spectroscopy analysis result of immunoprecipitation of whole-cell extracts using the Flag antibody with the indicated treatment.

| **Protein ID** | **Protein Name** | **Protein Score** | **Protein Mass（Da）** |
| --- | --- | --- | --- |
| Q96P20 | NOD-like receptor thermal protein domain associated protein 3 (NLRP3) | 3026 | 93200 |
| O75152 | Zinc finger CCCH domain-containing protein 11A (ZC11A) | 523 | 89931 |
| Q63ZY3 | KN motif and ankyrin repeat domain-containing protein 2 (KANK2) | 226 | 91916 |
| Q9UJV9 | Probable ATP-dependent RNA helicase DDX41 (DDX41 | 225 | 70477 |
| AOAVT1 | Ubiquitin-like modifier-activating enzyme 6 (UBA6) | 215 | 119207 |
| P31151 | Protein s100-A7 (S10A7) | 195 | 11578 |
| Q8IYT8 | Serine/threonine-protein kinase ULK2 (ULK2) | 188 | 114276 |
| Q13976 | cGMP-dependent protein kinase 1 (KGP1) | 152 | 76943 |
| Q14028 | Cyclic nucleotide-gated cation channel beta-1 (CNGB1) | 149 | 140502 |

Table S2. The information of DNA sequence used in the experiment.

| **Name** | | **Sequences (****5'-3')** |
| --- | --- | --- |
| NApt8 | TGCGTGTGTAGTGTGTCTGTAAAAATCGGGGGCGGGAGGTGGGGTACGGAGATCGGATTCTCTTAGGGATTTGGGCGG | |
| NApt8-1 | AAATCGGGGGCGGGAGGTGGGGTACGGAGATCGGATTCTCTTAGGGATTT | |
| NApt8-2 | TGCGTGTGTAGTGTGTCTGTAAAAATCGGGGGCGGGAGGTGGGGTACGGAGATCGGATT | |
| NApt8-3 | TAAAAATCGGGGGCGGGAGGTGGGGTACGGAGATCGGATTCTCTTAGGGATTTGGGCGG | |
| Seq-7 | GCAGGCAAGGCTCTACTGACCCGTTGCTTGATCGAC | |
| Seq-59 | ACTCCCACCTTTATTGAGGGCGGTGACGGGTTCCCTC | |
| Seq-333 | AGGGGGGACACCTAGATTGTTGGCCGTGCGGATACG | |
| Seq-1022 | AGGGGGGACATCTGGAGTGTTGGCCGTGCGGATACG | |
| SApt | GGCATCAAAGGGGGGAGGGCGGGTGGATTGGATGCCGA | |
| NASO | ATTGTTAGCAGGATTGCGGG | |
| SASON | GGCATCAAAGGGGGGAGGGCGGGTGGATTGGATGCCGAAAAAAAATTGTTAGCAGGATTGCGGGAAAAAATAAAAATCGGGGGCGGGAGGTGGGGTACGGAGATCGGATTCTCTTAGGGATTTGGGCGG | |
| circSASON | GGCATCAAAGGGGGGAGGGCGGGTGGATTGGATGCCGAAAAAAAATTGTTAGCAGGATTGCGGGAAAAAATAAAAATCGGGGGCGGGAGGTGGGGTACGGAGATCGGATTCTCTTAGGGATTTGGGCGG | |
| circSASON-mut | GATGGCGGTGTAGGGGCGGGGATCGGCGTTGGACGGGAAAGTTAACCCGAGGTTGCGGGAGGGGCATAAAAAATGGGGTGGGTCGGGAGATCGGATTCTCTTTGGGATTTGGGCGGGAAAAAAGGGGGG | |
| circSASO | GGCATCAAAGGGGGGAGGGCGGGTGGATTGGATGCCGATTTATTGTTAGCAGGATTGCGGG | |
| circSN | GGCATCAAAGGGGGGAGGGCGGGTGGATTGGATGCCGAAAAAAATAAAAATCGGGGGCGGGAGGTGGGGTACGGAGATCGGATTCTCTTAGGGATTTGGGCG | |
